# Theoretical prediction and shape-controlled synthesis of two-dimensional semiconductive Ni_3_TeO_6_

**DOI:** 10.1038/s41699-023-00412-1

**Published:** 2023-07-08

**Authors:** Javier Fernández-Catalá, Andrey A. Kistanov, Yang Bai, Harishchandra Singh, Wei Cao

**Affiliations:** 1 Nano and Molecular Systems Research Unit, University of Oulu, Oulu, FIN-90014 Finland; 2 Microelectronics Research Unit, Faculty of Information Technology and Electrical Engineering, University of Oulu, FI-90570 Oulu, Finland

**Keywords:** Two-dimensional materials, Photocatalysis, Theoretical chemistry

## Abstract

Current progress in two-dimensional (2D) materials explorations leads to constant specie enrichments of possible advanced materials down to two dimensions. The metal chalcogenide-based 2D materials are promising grounds where many adjacent territories are waiting to be explored. Here, a stable monolayer Ni_3_TeO_6_ (NTO) structure was computationally predicted and its stacked 2D nanosheets experimentally synthesized. Theoretical design undergoes featuring coordination of metalloid chalcogen, slicing the bulk structure, geometrical optimizations and stability study. The predicted layered NTO structure is realized in nanometer-thick nanosheets via a one-pot shape-controlled hydrothermal synthesis. Compared to the bulk, the 2D NTO own a lowered bandgap energy, more sensitive wavelength selectivity and an emerging photocatalytic hydrogen evolution ability under visible light. Beside a new 2D NTO with the optoelectrical and photocatalytic merits, its existing polar space group, structural specification, and design route are hoped to benefit 2D semiconductor innovations both in species enrichment and future applications.

## Introduction

Searching for 2D semiconducting matrix has been triggered by the invention of the high-performance monolayer transistor built on 2D MoS_2_ about a decade ago^1^. In comparison to the semi-metallic counterpart of mono-elemental graphene, the semiconducting chalcogenides, to which the MoS_2_ is grouped, are variable in species and versatile in functionalities. As extensions of the 2D MoS_2_ works, efforts have been poured into studies of naturally occurring at their single layer forms, such as 2D MoSe_2_, WS_2_, and WSe_2_^2,3^. More recent works emphasized on monolayered TMDs have shown diverse functionalities such as TaS_2_ displaying charge density waves^4^, NbSe_2_ for superconductivity^5^, and VSe_2_ for room temperature ferrormagnetism^6^. Beside the electronic, photonic, magnetic, optoelectronic, and electroluminescent applications^3,7–9^, the 2D chalcogen compounds are considered promising in solar light harvesting via photovoltaic^10^ and photocatalytic processes^11^_._

Previously, discovering of 2D materials has been realized via dedicated experimental approaches (exfoliation, wet chemistry, and CVD…)^12,13^, while more recent studies directed to computational design of 2D materials^14–16^. For instance, group VA 2D semiconductors, antimonene, arsenene, and bismuthene, for the very first time have been predicted using density functional theory (DFT)-based simulations^14^. DFT-based simulations have also played a substantial role in the developments of layered dichalcogenides^17^, transition metal carb(nitr)ides (MXenes)^18^, and dichlorides^16^. Inspired by the theoretical prediction by varying the elements within the atomic structures of known MXenes, ~70 potential MXenes^18^ have been successfully discovered and the new directions for their studies and functionalities are booming^19,20^. Recently, machine learning algorithms, another powerful tool for the prediction of new materials, have arisen and substantially enriched the 2D materials’ library^21^. The development of that tool is still ongoing, and material’s databases created with the help of machine learning are yet lack of many 2D samples. Despite computational progresses, realizations of the predicted structures are scarcely doable due to sophisticated arrangements of composition elements, stringent experimental conditions, ambient stability and moreover the discrepancies between predicted and experimentally determined properties^22^. The entire families of multi-component 2D materials are waiting to be discovered.

Many of the newly invented 2D materials have run far from the chalcogenide which debuted the trend of 2D semiconductors^23^. However, the metal chalcogenide-based 2D materials may shed light on materials invention route where the coordination features of chalcogens benefits bonding in compound formations. A specifically interesting element in chalcogenide family stays on the tellurium (Te). It owns 6 valence electrons with similar coordination capacities as other chalcogenide peers, while the metalloid character makes it capable of bonding to both oxidizing and reducing agents^24^. Yet, constructions of materials over the conventional metal-tellurium monolayers may end in surprising finding of new 2D species with semiconductor features. Along with the naturally occurring species, the 2D tellurides were engineered stably on NiTe_2_, SiTe_2_, SnTe_2,_ etc, free of oxygen^25,26^. However, there are a lack in computational and experimental studies based on 2D materials composed of the metal (M), Te and oxygen (O), being these bulk materials (MTO) very interesting for the scientific community for their magnetic, topological and electronic properties^27,28^. In this family of MTOs, one promising bulk material studied by the scientific community is Ni_3_TeO_6_ (NTO) semiconductor, due its magnetoelectric and photocatalytic properties^29,30^. However, a controlled synthesis is required to get the 2D form of the NTO. The preparation processes are different from the conventional route of solid state synthesis through which morphological controls are hardly carried out for the MTO family in general^31^. In addition, due to low melting point of Te, final products are associated with multiphases. For instance, the unwanted Co_3_O_4_ coexisted with the Co_3_TeO_6_ when synthesized via solid state reaction, as a result of low interaction between the reagents at high temperatures^32^. MTOs though equipped with aforesaid unique properties, remain at the bulk form and thinning them to slices is only conceptually available^33,34^. The scenario demands development of wet synthesis, such as hydrothermal method, that can control the coordination among M, Te, and O leading to 2D MTO for other energy-related applications^35,36^.

In this work, we computationally predict a stable 2D Ni_3_TeO_6_ and experimentally realize the predicted layered NTO structure in the thin nanosheet form. We show that the 2D NTO inherits the semiconductive nature from the bulk NTO, but bandgap size is narrowed with decreasing the dimensionality. The calculated properties are well in line with these determined experimentally. Owning to morphologic, electronic, and structural uniqueness, the outstanding pristine 2D NTO owns a lower electrical resistivity and better photoconductivity compared to the bulk counterpart. An emerging photocatalytic hydrogen evolution capability was found without any co-catalyst or scavenger under visible light irradiation. Beside adding a new compound to the 2D materials library, we hope this work will inspire forthcoming studies and application of NTO materials in various fields, such as magnetoelectric, topology and photonic applications, and open a new family of 2D MTOs.

## Results and discussion

### Computational design of 2D NTO

**Fig. 1:**
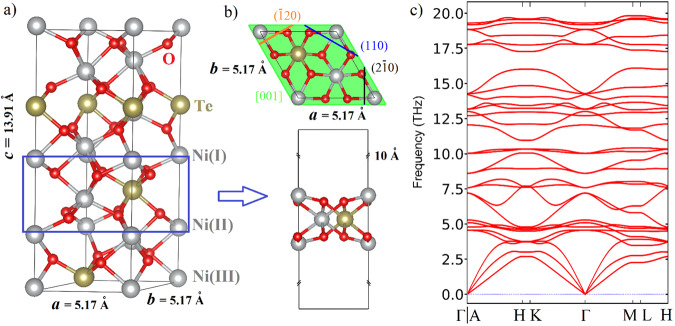
Theoretical prediction of 2D-Ni_3_TeO_6_. Computationally obtained unit cell of (**a**) a bulk and (**b**) 2D Ni_3_(I)TeO_6_ structure. **c** Phonon dispersion curves for the unit cell of 2D Ni_3_(I)TeO_6_.

First, we computationally designed the 2D NTO by slicing the trigonal bulk NTO of the space group # 146 (R3) and the lattice constants a = b = 5.17 Å and c = 13.91 Å (see Fig. 1a). The bulk NTO structure can be represented by three different Ni layers depending on their bonding conditions to neighboring atoms, namely Ni (I), Ni (II) and Ni (III), as denoted in Fig. 1a and the associated CIF files as the Supplementary Dataset 1. Correspondingly, three slices are composed in forms of Ni_3_(x)TeO_6_ (x = I-III)^37^. In the case of Ni_3_(I)TeO_6,_ slicing of the bulk NTO crystal into 2D slab is along the c-direction i.e., [001] zone axis. Planes intersecting along this zone axis are (110), ($\bar{1}$20), and (2 $\bar{1}$0) as denoted in Fig. 1b. Another two forms of Ni (II) and Ni (III) are depicted in Supplementary Fig. 1. The stabilities of optimized structures are assessed by lattice vibration in the output of phonon frequencies. As presented in Fig. 1c the transverse acoustic (TA), longitudinal acoustic (LA), and the out-of-plane z-direction acoustic (ZA) modes have positive frequencies and display the normal linear dispersion around the Γ point, which confirms a kinetic stability of model layer 2D Ni_3_(I)TeO_6_. The formation energy E_form_ was calculated as the difference between the energy of 2D NTO and energy sum of corresponding stable elementary substance Ni, Te and O^38^. A value of −0.44 eV/atom was obtained, confirming thermodynamic stability of the computationally designed 2D NTO^39^. Compared to the formation energy of the bulk counterpart (−0.889 eV/atom) (https://www.ctcms.nist.gov/~knc6/jsmol/JVASP-12261.html), a higher value from the 2D NTO denotes the metastable nature of the sliced phase. The thermostability of the 2D NTO was computationally evaluated by ab initio molecular dynamics simulation performed at 300 K. As shown in Supplementary Fig. 2, the total energy of the system remains unchanged at the selected time scale. The 2D phase is stable at room temperature. In fact, the 2D slice of Ni_3_(I)TeO_6_ is the only stable one when lowering the dimension from 3D to 2D for the bulk NTO. Another two forms are not stable according to phonon dispersion curves (see Supplementary Fig. 1 in Supplementary Information for more details). The 2D Ni_3_(I)TeO_6_ modeled by DFT has a trigonal space group and can be indexed with a space group of R3 symmetry with a slightly corrugated layer consists of NiO_6_–TeO_6_ honeycomb rings. The lattice parameters associated to optimized 2D NTO simulated layers is a = b = 5.17 Å (Supplementary Fig. 3 and CIF file in Supplementary Dataset 1). As it can be noticeable that Ni atoms stay on the surface of the 2D Ni_3_(I)TeO_6_ slab (Fig. 1b) due to broken Ni-O bonds (when slicing). Such an atomic configuration in the slices is posing possibly higher chemical activities than these of the bulk counterparts^40–42^, while the possible dangling bond of the Ni at the utmost layer requires functional groups to terminate the Ni to reach ambient stability.

### Characterization of synthetic 2D NTO

**Fig. 2:**
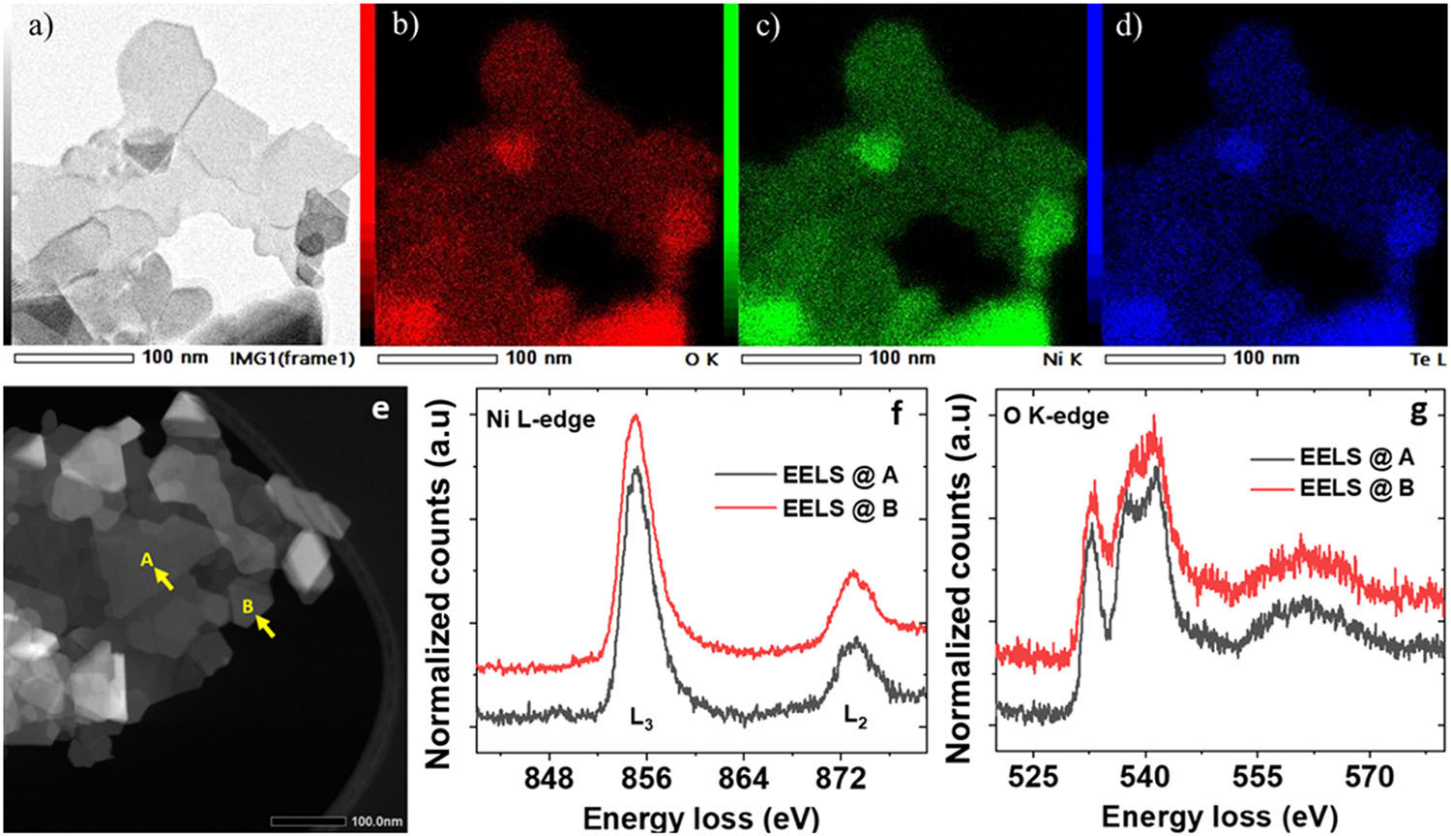
Chemical composition of 2D-Ni_3_TeO_6_. **a**–**d** STEM image and corresponding EDS mapping, **a** STEM images of layer- Ni_3_TeO_6_, elemental mapping images of **c** O, **d** Ni, and **e** Te elements. **e**–**g** STEM image and corresponding EELS spectra at two positions marked as A and B for (**f**) Ni L-edge and (**g**) O K-edge.

Following the density functional theory prediction of the dimensionally lowered and stable metal tellurate, the 2D Ni_3_TeO_6_ has been realized experimentally. To control the shape, hydrolysis of urea is implanted to reach the 2D materials’ structure in a one-pot hydrothermal synthesis^36,43,44^. The NTO nanosheets are clearly seen in the scanning transmission electron microscopy (STEM) image depicted in Fig. 2a. The elemental distribution of the NTO sheets is characterized by using energy dispersive X-ray spectroscopy (EDS) combined with the scanning TEM (STEM-EDS) in Fig. 2b–d. Presences of Ni (green), Te (blue), and O (red) are also confirmed. Moreover, the quantitative EDS analysis indicates that the atomic percentage of the elements Ni (32.4%), Te (11.1%), and O (56.6%) is very close to the stochiometric ratio as in Ni_3_TeO_6_^28^. Figure 2e–g show the electron energy loss spectroscopy (STEM-EELS) image and data associated with Ni L- and O K-edge EELS spectra for experimentally grown 2D NTO. The M-edge EELS of tellurium were plotted in Supplementary Fig. 4. Within the EELS data collection resolution limit, the results match with the structural analysis. The EELS data analysis includes data calibration with the published results for Ni L_2,3_ edge and O K-edge^45,46^. Together with oxidation state, Ni L_2,3_ edge data probes the unoccupied 3d states of Ni via electron transitions from spin orbit split levels 2*p*_*3/2*_ to 3*d* (L_3_ edge) and 2*p*_*1/2*_ to 3*d* (L_2_ edge). The prominent high-energy features near 855 eV and 872 eV are attributed to the existence of Ni^2+^ as expected for NTO. In the oxygen K-edge EELS spectra, a pre-edge feature centered at ∼532 eV results from transitions to unoccupied metal 3*d* orbitals that are hybridized with O 2*p* character (formally a 1*s* → 3*d* transition that gains dipole allowed intensity via O 2*p* mixing). Spectral intensity above the pre-edge (i.e., a broader set of overlapping bands spanning the 535–570 eV) is associated with states that have O 2*p* character hybridized with unoccupied metal 4*s/*4*p* and Te 5*pd* orbitals. The pre-edge intensity measures the strength of the covalency of metal-oxygen bonds, which further indicates the bond length similarly at both the selected sites and hence the geometrical coordination^47,48^.

**Fig. 3:**
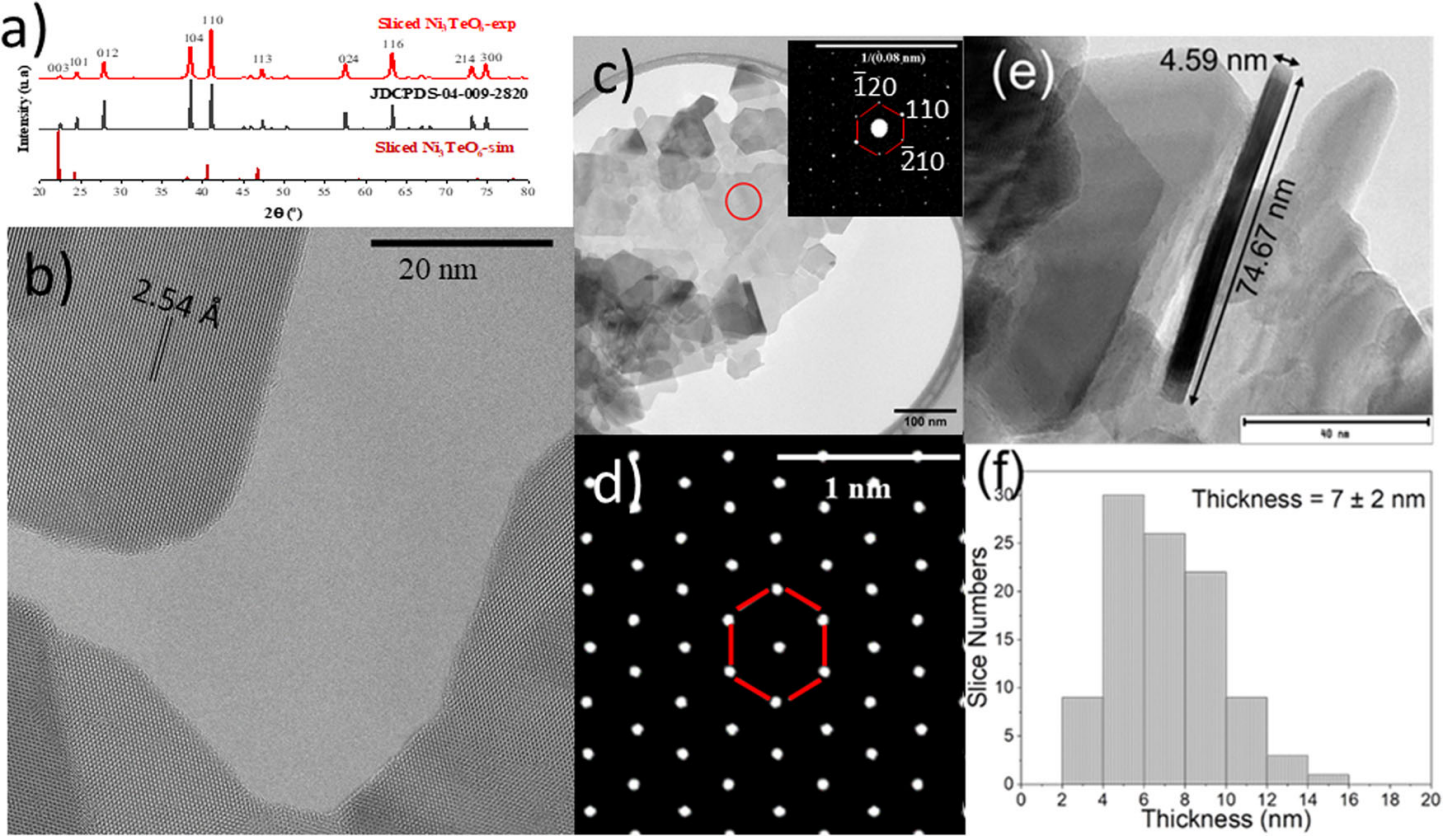
Structural and morphological analysis of 2D Ni_3_TeO_6_. **a** XRD patterns of the invented 2D Ni_3_TeO_6_, JDCPDS-04-009-282, and simulated sliced Ni_3_TeO_6_. **b** HR-TEM image of the NTO slab, **c** TEM image and its associated SAED pattern of as-prepared NTO, **d** Simulated STEM image of the monolayer-NTO, **e** a thin nanosheet with layered feature, and **f** average thickness of the nanosheets as statistically obtained.

The structural analysis of the of the invented 2D Ni_3_TeO_6_ is further investigated focusing on the structure and morphology of this material, using X-ray diffraction (XRD), transmission electron microscopy (TEM) and selected area electron diffraction (SAED) analysis. Following the characterization strategy of 2D materials^49–51^, beside the powder XRD for global structural investigation, the SAED patterns in the high-resolution TEM (HR-TEM) and DFT-simulated scanning tunneling microscopy (STM) images are employed to study 2D NTO. In Fig. 3a, the experimental XRD pattern (upper panel) was plotted with bulk NTO (JCPDS-04-009-2820) (middle pane) and simulated result of 2D NTO pattern (bottom panel). Generally, in Fig. 3a, experimental 2D NTO pattern have almost the same positions of diffraction peaks of Bulk NTO, instead of the simulated 2D NTO pattern where the preference orientation is [001], as it was observed computationally. The NTO nanosheets are randomly oriented to form agglomeration other than oriented sheets placed on surfaces, as shown in Supplementary Fig. 5. Due to this random orientation of the nanosheet agglomeration, the diffracted pattern shows similarity to the one of the bulk in a powder XRD analysis^52–54^. These at 2θ = 22.38°, 24.48°, 27.78°, 38.49°, 41.09°, 47.17°, 57.57°, 63.37°, 73.01°, and 74.62° are indexed to characteristic peaks from (003), (101), (012), (104), (110), (113), (024), (116), (214), and (300) lattice planes. However, the relative intensities of main peaks vary from these of 2D to the bulk counterpart. In the simulated patterns, (110)/(-1-10) has almost the same preference of orientation as the (104), different from the bulk case where (104) is the most predominate. The trend is even more obvious in the measured XRD where the former is more oriented with the largest intensity. This comparative XRD result indicates that the single-phased NTO is synthesized successfully. According to the relative peak intensities, 2D NTO shows (110) (-1-10) as the preferred orientation whereas bulk NTO exhibits (104) as the preferred orientation (Fig. 3a and Supplementary Fig. 6). When slicing the bulk along the [001] zone axis, the possible (110) plane turns out as depicted in Fig. 1b. Careful calculation of XRD peaks shows a tensile strain of 0.12% in 2D NTO compared to the bulk counterpart. The trend of strain agrees with the computational results. As detailed in Supplementary Fig. 3 associated with the tabulated values, unoptimized versus optimized layer structure of NTO indicate that Ni-O bond lengths are reduced after optimization, suggesting more planer orientation along the a-b directions.

The HR-TEM image of this 2D NTO in Fig. 3b shows a periodic atom arrangement of the NTO, demonstrating that the sample is crystalline. The lattice spacing corresponding to the observed lattice plane is 2.54 Å, which matches with (110) plane family of hexagonal structure, of the Ni_3_TeO_6_ material. The TEM-SAED pattern (Fig. 3b) reveals that the synthesized NTO sample presents a hexagonal lattice structure. The diffraction spots denote the same lattice spacing of 2.54 Å, corresponding to electron diffractions from the respective family of planes ($\bar{1}$20), (110), and ($\bar{2}$10) with a common [001] zone axis. The HR-TEM and SAED results are in line with these given by slicing route of Ni_3_(I)TeO_6_ in Fig. 1, by a DFT-based approach. This microscopic vision of the nanosheet and SAED pattern is also consistent with the DFT-simulated scanning tunneling microscopy image of the 2D slice of Ni_3_(I)TeO_6_ (Fig. 3d). These experimental results demonstrate a stable hexagonal 2D NTO nanosheet has been synthesized, as it was predicted by the 2D-NTO DFT-model.

We further investigated the structural details of the thinned Ni_3_(I)TeO_6_ (Supplementary Fig. 7). The nanosheet form with layered features is seen on a 4.6 nm thin nanosheet where alternatively turn out in Fig. 3e, indicating the crystallinity of the samples. An average thickness of 7 nm is obtained for the 2D NTO according to a statistic survey of ~100 pieces in Fig. 3f. As it has been mentioned above, according to our DFT results, the 2D slice of Ni_3_(I)TeO_6_ is the only possible one. To confirm the layered structure of the Ni_3_(I)TeO_6_, we selected a thick NTO sheet and analyzed the sample (with rotation of 90 degree) in the tomography mode of TEM and SAED (Supplementary Fig. 8a–d). TEM images of the sample (Fig. 3c and Supplementary Fig. 8a) clearly visualize it in layers together with groupings of various layers forming nanosheets. Moreover, as per the obtained SAED image (Supplementary Fig. 8c), the sample present the hexagonal structure with the lattice spacing of 2.54 Å corresponding to the family of planes (110). After a rotation of 90 degree (Supplementary Fig. 8b), the morphology of the sample has been changed from the plate like structure to stack. Additional evidence of this change is shown in Supplementary Video 1, where a series of images with different rotation angles along the x-axis clearly demonstrate the 2D morphology of the invented 2D NTO. The sample in vertical position was analyzed by SAED obtaining the plane (001) (Supplementary Fig. 8d), which further confirms that the sample synthesized present the stable [001] zone axis for 2D NTO, matching well with the XRD results and computational predictions. Low magnification images (Supplementary Fig. 8a, b) show a single NTO nanosheet together with groupings of various layers forming nanosheets. The multilayer feature is obviously in Supplementary Video 1 where nanosheets can be visualized as stacks of the 2D layers. The morphologic determinations here are in line with the stacked layer feature which was also seen at the bottom left corner in Fig. 3b.

The synthetic 2D Ni_3_(I)TeO_6_ is stable in ambient conditions. The TEM image recorded in a different position of sample 2D NTO in Supplementary Fig. 9a shows the same morphology as the one in Fig. 3, indicating that the sample is homogeneous. However, in the Fig. 2c and Supplementary Fig. 9 there are different shapes of nanosheets, for example, rhombus, trapezoid and polygon. This fact is due the synthetic method (hydrothermal methodology) used in the preparation. In this methodology usually the small sheets tended to agglomerate, which could grow together and contribute to the growth of big nanosheets^43,44,55^. Furthermore, the sample preparation for TEM measurements vaporizes water and solid flakes aggregate. To study the stability of the 2D NTO the sample was analyzed by TEM and HR-TEM around 3 month and one year later, see dates of the analysis (Supplementary Fig. 9). The TEM images Supplementary Fig. 9c, e show that the sample present the same morphology, besides in HR-TEM (Supplementary Fig. 9d, f) the material presenting crystallinity. This fact indicate that the sample is stable in ambient conditions. It is also worthy noting that the shape-controlled synthesis to form nanosheet is well repeatable. Under the same preparation condition, the product owns the same morphology and crystallinity as demonstrated by TEM and HR-TEM images of the as-prepared sample in another synthesis (Supplementary Fig. 9b, c).

### Optical, electronic, and magnetic properties of 2D NTO

**Fig. 4:**
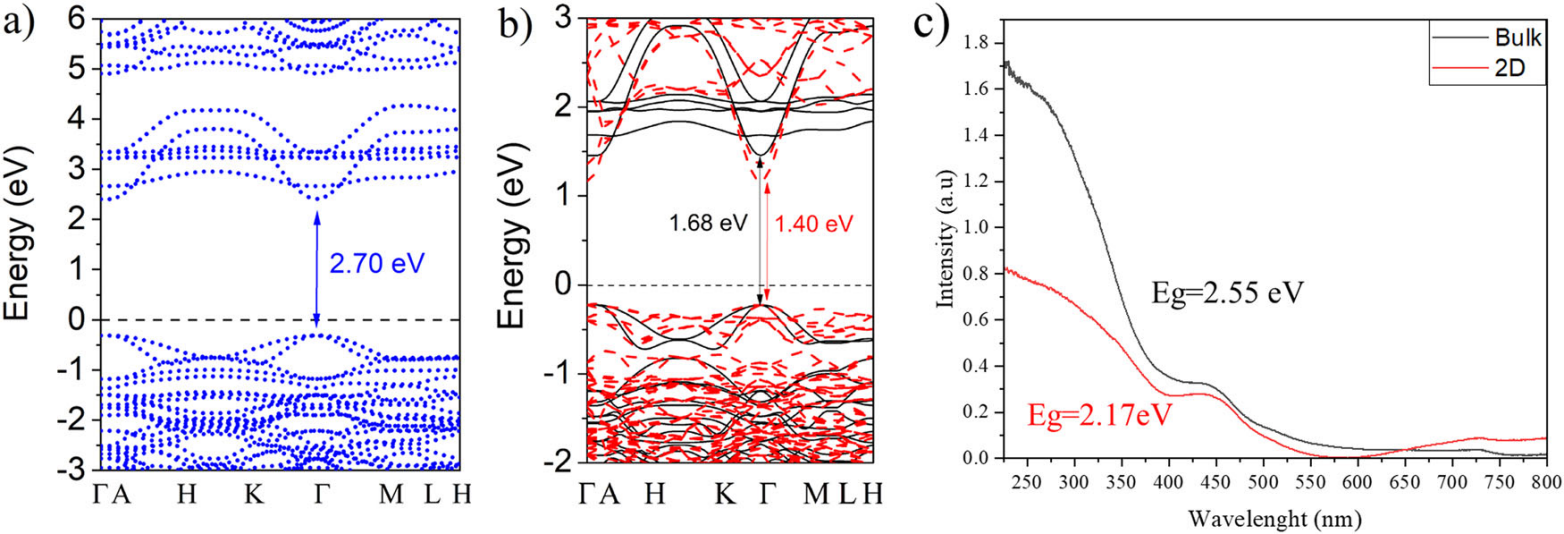
Optical properties and band structure of 2D Ni_3_TeO_6_. **a** DFT predicted band structure of the 2D Ni_3_TeO_6_ calculated using HSE functional. **b** DFT predicted band structure of the 2D (red dashed line) and bulk (black line) NTOs calculated using GGA + U functional. **c** UV-Vis spectra of the 2D and bulk NTOs.

Electronic structures of the bulk and 2D Ni_3_TeO_6_ are studied computationally and spectroscopically. DFT results, shown in Fig. 4a, suggest that 2D NTO is a semiconductor with a bandgap of ~2.70 eV (HSE functional, more details in Supplementary Fig. 10 and Supplementary Information). In addition, DFT calculations also suggest that the bandgap of NTO narrows by ~0.28 eV with the decrease in its dimensionality (Fig. 4b). It should be noted that the HSE method may overestimate values of the bandgap size obtained experimentally^56,57^. At the same time GGA approach may underestimate values of the bandgap size obtained experimentally. Therefore, it can be said that the experimental value of the band gap size of 2D NTO should be within the range from 1.68 eV (GGA) to 2.7 eV (HSE). Concerning 2D NTO spectroscopic analysis, Fig. 4c shows the UV-Vis spectra of the sliced NTO, where the synthesized 2D NTO presents an abrupt cutoff indicating that it is a semiconductor^58^. The absorption spectra of 2D NTO lies in the range of 200–500 nm. Observed second band refers to three-spin allowed *d–d* transitions from the Ni^2+^ ions in NTO^29,59^. The bandgap of the layered NTO, determined by Tauc plot analysis (Supplementary Fig. 11), is found to be 2.17 eV, slightly lower than 2.55 eV of the bulk NTO which was also synthesized in a hydrothermal condition. The trend is in line with the one given by DFT calculations as seen in Fig. 4b. To compromise computational efficiency and accuracy, the band structures of 2D and bulk are compared using GGA + U approach as it is also giving high precision results^56^. Although, the bandgap of layered materials is usually reduced as the number of layers increases, there are few exceptions, such as Mica^60^. Moreover, a wide-bandgap materials with the unique property of exhibiting bandgap narrowing with the decrease in the number of layers^58^ or the overall size of the materials^61^. It is worth noting that the trend of bandgap energies when lowering the NTO dimension cannot be grouped or compared to these of the layered crystals^58,60,61^. The stable 2D NTO layer is not a part of the 3D NTO, yet not constituting layered stacks via van der Waals force to form the bulk crystal.

To better understand the physical nature of a decrease of a bandgap energy of NTO with decreasing the dimensionality, the partial densities of states for 2D and bulk NTO are plotted in Supplementary Fig. 12. It is found that in case of 2D NTO was calculated, the bandgap decreases happening due to a stronger hybridization between Ni-d and O-p states and flattering of conduction bands, compared to that of bulk NTO. Moreover, the reduction in bandgap observed in the 2D NTO may also be due to change of boundary conditions from bulk to thin slides where changes of tensile strains are associated for the in- and out- planes of the material^62,63^. This is evidenced in shifts in XRD peaks towards lower 2θ angles from the above XRD analysis and also zoomed XRD patterns as shown in Supplementary Fig. 13.

**Fig. 5:**
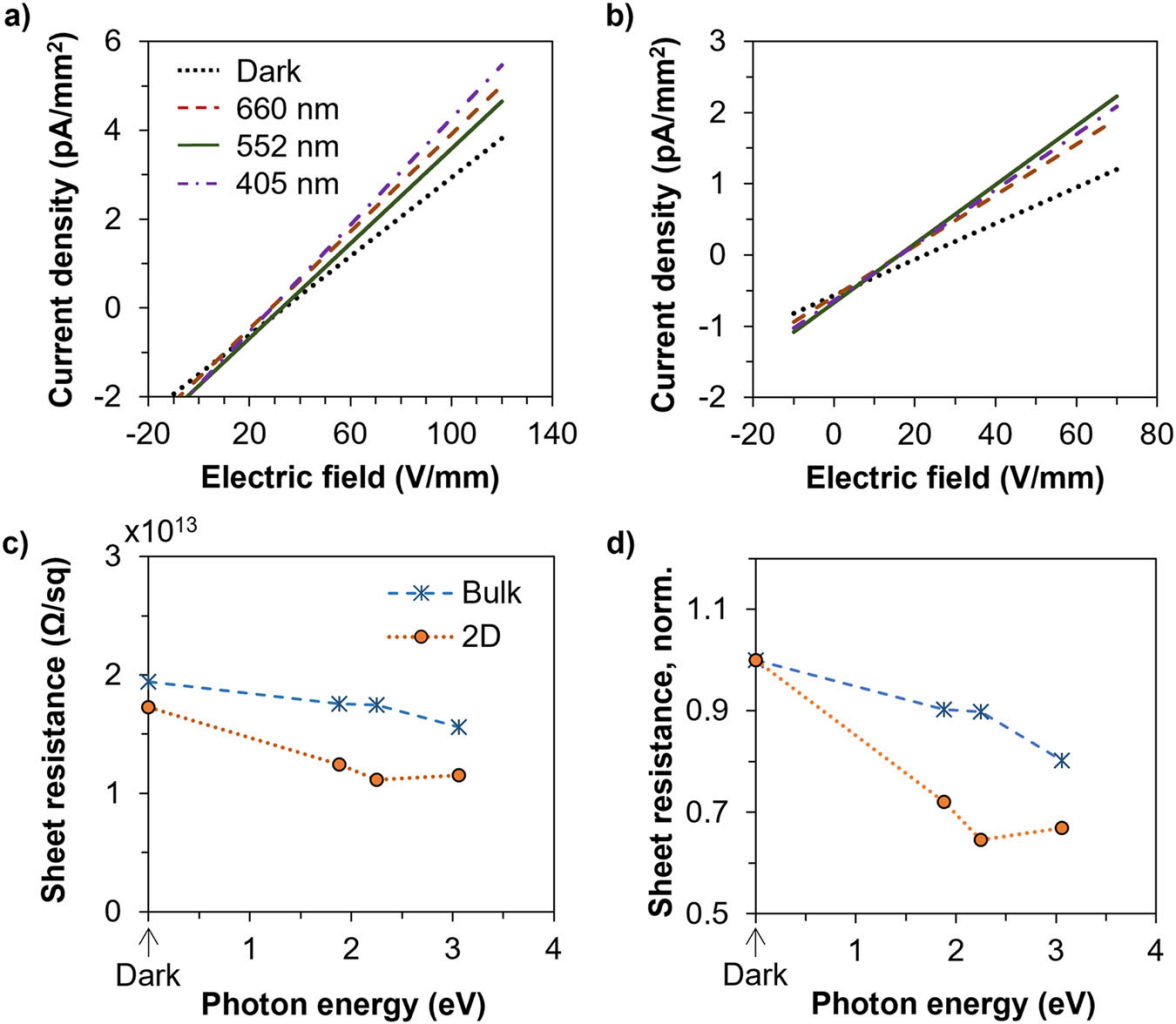
Photoelectric properties of the 2D Ni_3_TeO_6_. Dependence of current density on bias electric field for (**a**) the bulk and (**b**) the 2D materials measured in the dark and under laser beams with different wavelengths (660 nm, 552 nm and 405 nm); **c**, **d** Dependence of (**a**) sheet resistance and (**b**) normalized sheet resistance (with the values in the dark as ‘1’’) on photon energy of the laser beams for the bulk and 2D materials.

The electronic structure and low-dimensional feature of the 2D NTO denotes possible differences of electric properties compared to these of the bulk form. Figure 5 reveals the behaviors of electric conductivity of the bulk and 2D materials. For both types of the materials, photoconductivity could be observed with increased current density under the same electric field but when exposed to lasers with decreasing wavelengths (Fig. 5a, b). In the dark as well as under corresponding lasers, the sheet resistance of the 2D form was lower than that of the bulk form, implying a better conductivity of the 2D materials than that of the bulk counterpart (Fig. 5c). This is another evidence reflecting the previous discussions of the lowered band gap of the 2D form compared to the bulk form. The lowered band gap resulted in an increased photoconductivity, as shown in Fig. 5d where the sheet resistance of the 2D materials decreased by more than 30% when subject to illumination while the decrease was less than 20% for the bulk materials. In addition, the sharper decrease of the sheet resistance (increase of conductivity) under photon energy of <2.5 eV exhibited by the 2D form compared to that of the bulk form was expected according to Fig. 4c.

Supplementary Fig. 14 assesses the magnetic properties of the bulk and 2D NTO powders by measuring their permeabilities at room temperature. A ferrite reference sample was measured under the same condition for comparison. As the powders were deposited on a plastic substrate, the permeability of the clean substrate without any powder deposition was also measured. The reference sample and the plastic substrate had the same dimensions and thus the results should be directly comparable with each other. It is clear that the bulk and 2D NTO did not show magnetic properties given the negligible and flat permeability curves across the measurement frequency range. This was believed to be due to the transition temperature was lower than room temperature, which has been proven elsewhere^37,64,65^.

### H_2_ production functionality of 2D NTO

**Fig. 6:**
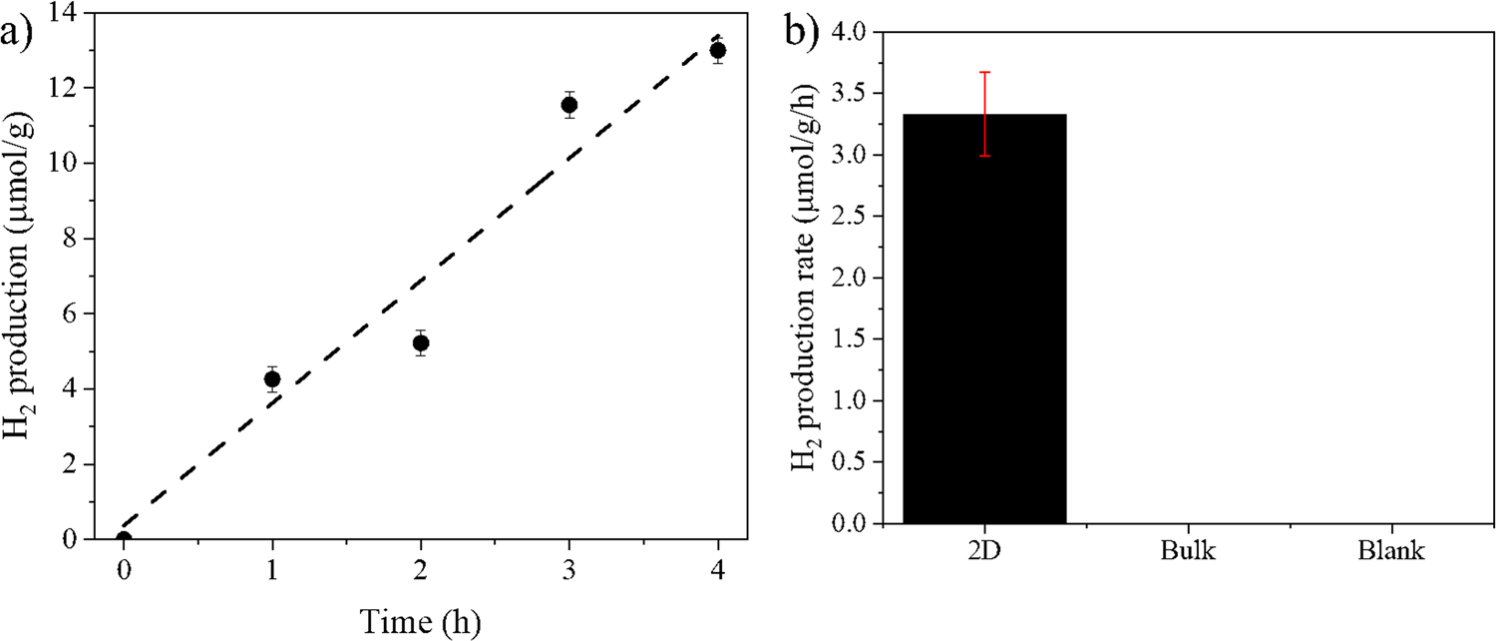
Photocatalytic H_2_-production activities of 2D Ni_3_TeO_6_. **a** Time course of photocatalytic hydrogen evolution on 2D Ni_3_TeO_6_ (dash line correspond to the line fitting, indicating the production of H_2_ with respect to the time) and **b** comparisons among photocatalytic activities of sample 2D NTO, bulk NTO material, and blank test without catalyst (Blank). Bulk NTO and blank test do not show photocatalytic activity in hydrogen evolution. The error bars in figures represent standard deviations calculated from 3 measurements.

We assessed possible applications of the invented 2D Ni_3_TeO_6_ for environmental remediation issues, such as water splitting, as it possesses all necessary characteristics to be an outstanding material in this application such as a moderate bandgap energy, large surface area, and high environmental stability. During photocatalysis, the catalysts are exposed to aqueous ambience, light irradiances and radicals generated from photocatalysis which may lead to photocorrosion and ruin the photocatalysts^66^. For this reason, we evaluated the photocatalytic activity towards HER following the reaction time and calculate the average HER rate. The 2D NTO nanosheets are found photocatalytically active in hydrogen evolution reaction under visible light irradiation (see Supplementary Fig. 15). The time course (see Fig. 6a) of the HER demonstrates its photocatalytic activity along with the stability of the slabs in an aqueous ambience. A lineal increase of H_2_ production is associated with time of reaction. An HER rate of 3.3 ± 0.3 µmol/g/h is obtained under low-power white LED irradiation (nominal power of 0.495 W) without any co-catalysts or scavenger with respect to the inactive bulk NTO as shown in Fig. 6b. The activity of the photocatalyst does not decrease after ten cycles (see Supplementary Fig. 16), indicating that 2D-NTO system is stable under condition of reaction. The thickness impacts on HER activity were investigated. Supplementary Fig. 17 shows the TEM images and the photocatalytic activity of NTO with a different thickness as a result of parameter tuning in the calcination process (see experimental process). Compared with the rather inert bulk NTO (~49 nm thick particles), both 2D NTO samples are photocatalytically active in hydrogen evolution reactions. A thinner catalyst results in a higher HER rates, due to larger surface areas per unit mass compared with the thicker counterpart.

The NTO nanosheets own superior photocatalytic activity in H_2_ production over the well-known 2D matrixes, e.g. the graphene, g-C_3_N_4_, MoS_2_, with low or negligible catalytic activities under visible light^67^. In fact, the unique HER ability on the synthetic 2D NTO with moderate bandgap energy and environmental stability is not seen on those intensively-studied pristine catalysts of TiO_2_^68^, BiVO_4_^69^, and layered MoS_2_^70^, see Supplementary Table 1. To test this effect with the same reaction condition, Supplementary Fig. 18 shows the photocatalytic activity of 2D-NTO, exfoliated MoS_2_, bulk MoS_2_ and P25. The catalytic test show that the NTO synthesized in this work present the best catalytic activities compared with other semiconductors. Different from the intrinsic HER capability of the 2D-NTO, co-catalyst, heterojunction constructions, or sacrificial agents are typically needed to active those compounds for hydrogen evolution under visible light. Thus, the stable 2D NTO is promising for photon-based applications.

To study possible photocatalytic mechanism of the semiconductor 2D NTO, scavenger tests were carried out by using the methanol (10% vol.) and AgNO_3_ (0.1 M) solutions to quench photoinduced h^+^^71,72^ and e^-^^73,74^ respectively. The HER experiments were performed in the same conditions as these for pristine sample (~7 nm thickness). Supplementary Fig. 19 shows that the incorporation of methanol increases the catalytic activity of 2D NTO while almost no hydrogen could be detected after incorporation of AgNO_3_. This fact indicates the important role of electron to perform the H_2_ production, since the electron is necessary in the water catalysis to reach H_2_.

The photocatalytic activity is affected by large number of factors^75^. First, the moderate bandgap energy of the 2D NTO enables visible light absorption which creates electron-hole pairs, as the first step of photoexcitation of semiconductor photocatalysts. Second, the photoinduced electron and hole play a key role in water catalysis, with the former one crucial to the current 2D system. As indicated by results of scavenger tests, a prolonged duration of the e^-^ after quenching the holes widens the time window of HER where hydrogen gas is formed by reduction and combination radicals of hydrogen atoms^70^. On the contrary, quenching the electrons results in undetectable amount of H_2_ but reductions of Ag^+^ to Ag nanoparticles as shown in Supplementary Fig. 19. Third, surface chemistry of photocatalysis benefits atomic configurations within the 2D metal tellurate. Different from the well-packed structure in the bulk NTO, though partially broken, the Ni-O bonds remains in the sliced and stabilize the 2D structure. The nickel components (NiO or metallic form) are well used as co-catalyst or catalyst in water splitting^40,41,76^. Therein water molecules tend to be adsorbed on Ni atoms which are acting as the active sites. The presence of Ni atoms on the slab surfaces in Fig. 1 resembles the one in the Ni@NiO/NiCO_3_ core-shell structures, where the adsorption tendencies have been computationally verified^77^. The interaction of the 2D NTO with aqueous environment facilitates the current photocatalysis. As shown in Supplementary Fig. 20, the -Ni(OH)_2_ feature was notified on the surface of the as-prepared 2D slab and the one after photocatalysis. This functional group of -OH terminates the Ni atoms to protect the 2D structures in the water ambience, in line with assumption in the theoretical part. The appearance of the hydroxide indeed is in favor of water splitting thanks to its role in oxygen evolution reaction (OER)^78^ to interact with the ·OH radicals produced in water oxidization. The overall chemical species are kept for the 2D slab, further denoting the high recyclability of this material for photocatalysis. Last but not the least, the low dimensionality is another important factor since the thin nanosheets possess more surface-active sites than the bulk counterparts^79,80^. The photoinduced e^-^ can migrate easier from inside of the slabs to the surfaces, as proved by a better photoconductivity from the 2D system in Fig. 6 and Supplementary Fig. 17. Later, they get involved in the HER through which hydrogen molecules were formed eventually.

In conclusion, a new, stable and semiconductive 2D material of Ni_3_TeO_6_ in the morphology of nanosheets was invented. The DFT-predicted 2D slab, sliced from the bulk counterpart in the metal tellurate family, was experimentally synthesized and proved stable. The morphological and electronic properties of the synthetic 2D NTO well match these computationally predicted results, bolstering the uniqueness of the prediction-to-realization route of materials innovation. Furthermore, the new material presents interesting electric and photonic properties due to semiconductive and moderate bandgap (2.17 eV). The pristine 2D NTO nanosheets are found moderate photocatalytically active in hydrogen evolution reaction without co-catalyst and scavenger under visible light. The magnetic properties of the 2D slabs are also evaluated and found nonferromagnetic in room temperature. Along with potential utilities in the photocatalysis and electronics of the invented material itself, this work is hoped to debut explorations the manmade 2D tellurate for various applications. The route combining theoretical and experimental endeavors is also hoped to inspire the enrichment of 2D tellurium materials and their potentials in overall materials science and functionalities.

## Methods

### Density functional theory calculations

The structures of 2D NTO were designed based on the geometry of primitive unit cell structures of bulk Ni_3_TeO_6_ (Fig. 1a) available in the Materials Project database (ID mp-19448)^81^. For the unit cell of each obtained structure (Ni_3_(I)TeO_6_, Ni_3_(II)TeO_6_, Ni_3_(III)TeO_6_) a geometry optimization was performed, and the stability of those structures was verified by calculating phonon dispersion spectra. Based on those simulations a stable modification of 2D NTO (Ni_3_(I)TeO_6_) was selected.

All calculations were performed using the plane-wave method as implemented in the Vienna Ab initio Simulation Package (VASP)^82^. The Perdew−Burke−Ernzerhof (PBE) exchange−correlation functional under the generalized gradient approximation (GGA)^83^ was used for the geometry optimization calculations. To achieve the accuracy in the electronic structure simulations, the calculations were conducted using several functionals which are PBE GGA, GGA with the Hubbard U correction^84^, and the hybrid Heyd−Scuseria−Ernzerhof (HSE)^85^. The optimization was stopped once the atomic forces and total energy values were smaller than 10–4 eV/Å and 10–8 eV. The first Brillouin zone was sampled with a 6 × 6 × 1 k-mesh grid and the kinetic energy cut-off was set to 520 eV. The periodic boundary conditions were applied for the two in-plane transverse directions. To avoid artificial interactions in the considered supercells, the vacuum depth of 20 Å was introduced to the direction perpendicular to the surface plane. Phonon dispersion spectra was calculated for the 3 × 3 × 1 supercell using finite displacement approaches with the atomic displacement distance of 0.01 Å accessible in the Phonopy code^86^ associated with VASP. The Tersoff-Hamann approach^87^, available in VASP, was applied to simulate scanning tunneling microscopy (STM) images. The Ab-Initio Molecular Dynamics (AIMD) simulation was conducted with Vienna Ab-initio Simulation Package (VASP)^82,88^. The calculation was run with NVT ensemble at 300 K for a time duration of 8 ps and the time step of 1 fs. The temperature was controlled by the Nosé-Hoover thermostat with an effective mass equal to zero. The Perdew-Burke-Ernzerhof (PBE) pseudopotentials^89,90^ and the Γ centered k-point grid were used. The convergence criterion was set to 10–5 eV and the energy cutoff was 520 eV for a 4 × 4 supercell.

### Reagents and materials

Nickel nitrate (Ni(NO_3_)_2_⋅6H_2_O, 98%, Alfa Aesar), telluric acid (H_6_O_6_Te, 98%, Sigma-Aldrich), urea (NH_2_CONH_2_, 99.0–100.5%, Sigma-Aldrich), absolute ethanol (EtOH, 99.5%, ETAX), and deionized water were used in the present work. All reactants were used as received, without further purification.

### Materials preparation

Synthesis of 2D nanosheets NTO: 2D NTO materials was prepared by one-pot hydrothermal synthesis. For the synthesis appropriate amounts of reagents, Ni(NO_3_)_2_·6H_2_O, and H_6_O_6_Te, were mixed in a stoichiometric ratio (3:1, respectively) in DI water (60 ml). To obtain this material, first a solution A was prepared by mixing the adequate amount of H_6_O_6_Te (0.26 g) and urea (0.4 g) in deionized (DI) water (30 mL). Solution A was stirred vigorously (5 min) to ensure a complete dissolution of the reactants. Then a solution B was prepared via mixing the adequate amount of Ni(NO_3_)_2_·6H_2_O (1 g) in DI water (30 mL) under vigorous stirring for 5 min. This solution was mixed and transferred quickly to a teflon-lined stainless-steel autoclave (100 ml of capacity, TEFIC hydrothermal synthesis autoclaves). The hydrothermal synthesis was performed at 180 °C for 12 h. Urea hydrolysis was applied to control the morphology of the material and form nanosheets, in line with the Bi_2_O_2_CO_3_ nanosheet synthesis^43^. The product obtained was centrifuged at 5000 rpm to collect the material in solid form. The product was washed three times with DI water (8 ml) and three times more with EtOH (8 ml) and dried at vacuum overnight. The solid obtained was calcined at 600 °C (2 h). NTO obtained with hydrothermal synthesis assisted by urea hydrolysis were namely as 2D NTO. The synthesis was repeated to ensure the repeatability. For comparison purposes, we also synthesized the same compound in the bulk form (Bulk NTO) in the hydrothermal method. To study the effect of the thickness of the NTO in the photocatalytic activity, the NTO was calcined at different ramps until achieve 600 °C for 2 h.

### Materials characterizations

Powder XRD patterns at room temperature were performed with Rigaku SmartLab 9 kW equipped with five-axis θ-θ goniometer and 1D solid-state detector and scintillator using Co-Kα (λ = 1.79 Å, 40 kV, 135 mA) radiation with scanning rate of 4°/min, and step of 0.02° in the 2θ range 20–80°. The powder samples are incorporated in standard glass holders to perform the analysis. TEM images coupled with EELS and energy dispersive spectroscopy (EDS) mapping were carried out using a JEOL JEM-2200FS EFTEM/STEM. The samples were suspended in ethanol to obtain a dispersion before drop-casting them on a copper grid and then placed in the measurement chamber. The tomographic tilt-series were acquired by transmission electron microscopy (TEM) and a high tilt-holder of JEOL. Images were recorded every 1° in the tilted angle range of −73 to +20°. UV-Vis spectra were recorded on a Shimadzu UV-2600 spectrophotometer. The absorbance of the materials was measured in the range of 200 nm–800 nm using a solution of powder of each sample with distillated water (10 mg in 8 mL). Distillated water is used as background. The surface sensitive technique of X-ray photoelectron spectroscopy was performed with Al-Kα using Thermo Fisher Scientific ESCALAB 250Xi XPS System. Energy calibration of the XPS was performed by using C 1 s peak at 284.8 eV.

### Photoconductivity and magnetic measurements

Powders of the 2D and bulk materials were deposited on glass and plastic substrates. The methodology used was drop coating using a dispersion of bulk NTO and 2D NTO (50 mg) in EtOH (5 mL). The drops deposited on sapphire substrates was dried at 80 °C. For the photoconductivity, a pair of silver ink strips was then coated on each sample on glass substrate to form a transverse electrode configuration for conductivity measurement. Standard J-E (current density-electric field) curves were collected from a source meter (Model 2450, Keithley, USA) in the dark and under laser illumination. Monochromatic lasers (OBIS LX/LS series, Coherent, USA) were used as the light sources. For the magnetic properties, permeabilities of the samples on plastic substrates were measured with an RF impedance/material analyzer (E4491A, Agilent, USA) at room temperature in the frequency range of 1 MHz–1 GHz.

### Catalytic test

The photocatalytic activities of the materials designed and prepared were measured by pure water splitting under visible light irradiation without any scavenger on Perfect Light PCX50B photoreactor. The white LED (λ > 420 nm), employed as the irradiation source here, has a nominal power of 0.495 W. The photocatalytic activity of H_2_ production was performed in a quartz bottle with height of 90 mm, diameter of 35 mm and total volume of 68 mL. 2D NTO photocatalyst (5 mg) was suspended in DI water (25 mL). Before the illumination with visible light, the dispersion was stirred for 30 min in dark. The prepared solution was exposed to light for 4 h at room temperature, each one hour the gas generated in the bottle has measured. The H_2_ generated was measured by Agilent Micro 490 GC gas chromatograph (GC) equipped with a Molesieve 5A column, as was previously reported^45^. To comparation bulk NTO material was also analyzed in the same conditions. Additionally, a blank test without catalyst was performed under the same experimental conditions as the catalytic tests and no catalytic activity was detected in the absence of photocatalyst. To study the stability of the 2D-NTO under reaction condition, cyclability tests were carried out. 2D NTO was measured 10 times reusing the same material under the same conditions explained above. An Agilent 8860 GC was employed to double-check and quantify the evolved hydrogen.

### Supplementary information

Supplementary Information

Supplementary Dataset 1

Supplementary Video 1

## Data Availability

Data generated and analyzed in this work are included in the article and its Supplementary Data. They are also available in zenodo repository at https://zenodo.org/record/7643292.
