# Supplementary material for: Theoretical prediction and shape-controlled synthesis of two-dimensional semiconductive Ni3TeO6
Source: NPJ 2D Mater Appl. 2023 Jul 8;7(1):48. doi: 10.1038/s41699-023-00412-1 (PMC11041737; doi:10.1038/s41699-023-00412-1)
Supplement: Supplementary file 1 — Supplementary Information [file 41699_2023_412_MOESM1_ESM.pdf]

Supplementary Information

Theoretical Prediction and Shape-controlled Synthesis of Two-dimensional Semiconductive Ni_3TeO_6

Javier Fernández-Catalá[1+], Andrey A. Kistanov[1+], Yang Bai[2], Harishchandra Singh[1], and Wei Cao[1] **

[1]Nano and Molecular Systems Research Unit, University of Oulu, FIN-90014, Finland

[2] Microelectronics Research Unit, Faculty of Information Technology and Electrical Engineering, University of Oulu, FI-90570 Oulu, Finland

E-mails: harishchandra.singh@oulu.fi wei.cao@oulu.fi

[+] These authors contributed equally to this work.

Table of Contents

Supplementary Figures……………………………………………………………….2

Supplementary Table ……………………………………………………………...15

Supplementary References………………………………………………………….16

Supplementary Figures

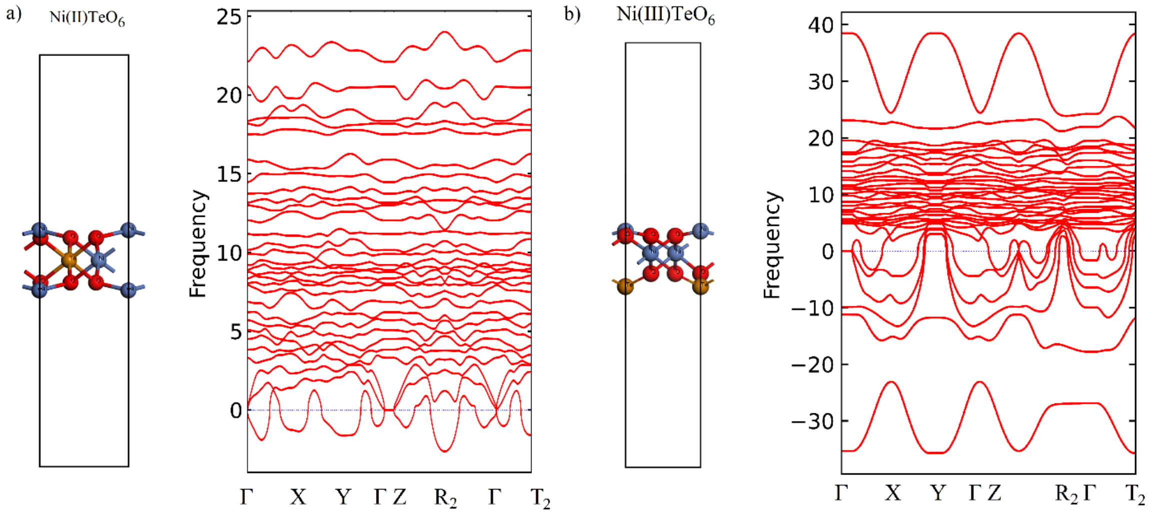

Supplementary Figure 1. *Theoretical prediction of two unstable 2D-Ni_3TeO_6 unit cells.* Computationally obtained unit cell of 2D (a) Ni(II)TeO_6 and (b) Ni(III)TeO_6 and phonon dispersion curves for these unit cells. The instabilities of the optimized Ni(II)TeO_6 and Ni(III)TeO_6 structures are shown via phonon dispersion curves, where negative phonon frequencies exist.

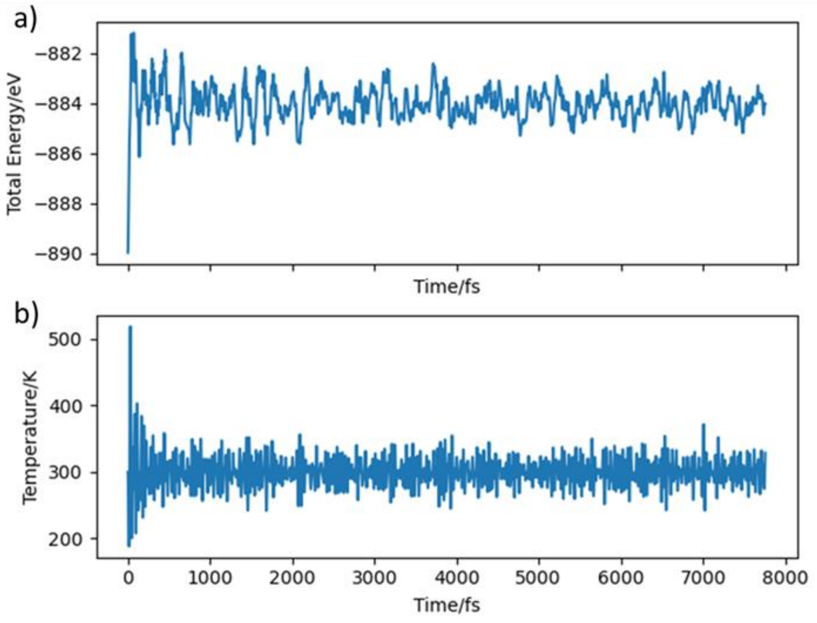

Supplementary Figure 2. *Theorical stability test of 2D NTO systems using MD simulation.* Evolution of the a) relative potential energy (eV) and temperature b) of the 2D NTO systems in MD simulation during an 8 ps period of simulation time. A time step of 1 fs was employed.

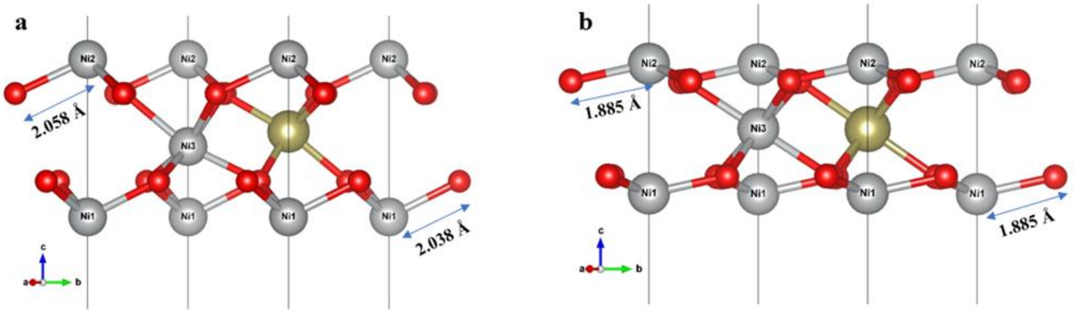

Bulk	x	y	Z
Ni1	0.000000	0.000000	0.217780
Ni2	0.000000	0.000000	0.507620
Ni3	0.666670	0.333330	0.347390
Te1	0.333330	0.666670	0.374930
O1	0.628880	0.669410	0.283380
O2	0.330590	0.959470	0.283380
O3	0.040530	0.371120	0.283380
O4	0.992320	0.635680	0.444780
O5	0.364320	0.356640	0.444780
O6	0.643360	0.007680	0.444780

2D	x	y	z
Ni1	0.000000	0.000001	0.474983
Ni2	0.000003	0.000003	0.584720
Ni3	0.666662	0.333329	0.529840
Te1	0.333332	0.666662	0.529868
O1	0.637051	0.650747	0.489538
O2	0.349252	0.986305	0.489538
O3	0.013696	0.362948	0.489539
O4	0.013594	0.650707	0.570161
O5	0.349297	0.362892	0.570161
O6	0.637114	-0.013593	0.570161

Supplementary Figure 3. *Optimization of layer structure of NTO.*

Un-optimized (a) vs optimized (b) layer structure of NTO and their corresponding Wyckoff sites for all atoms.

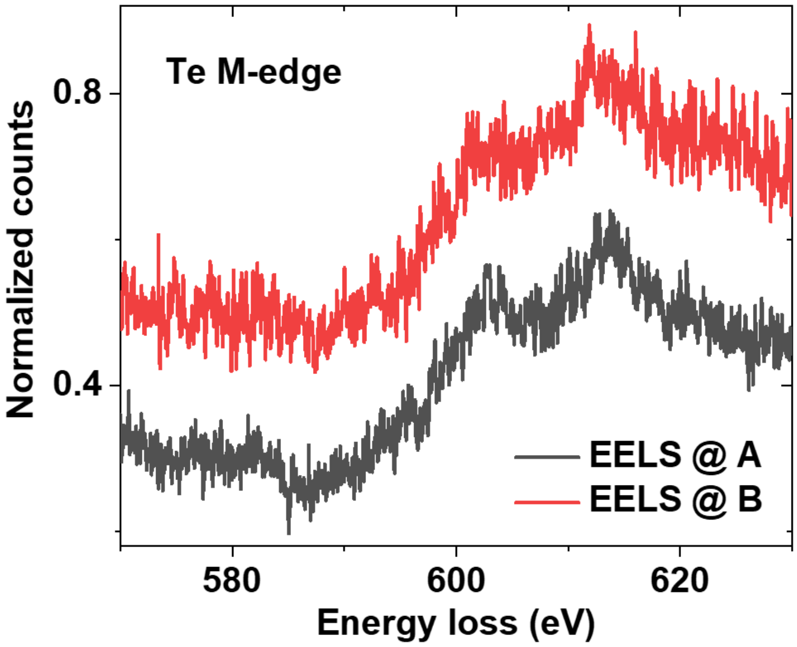

Supplementary Figure 4. *Presence of Te element in 2D NTO synthetic materials.*

EELS spectra at two positions marked in STEM Figure 2 (e) as A and B for Te M-edge.

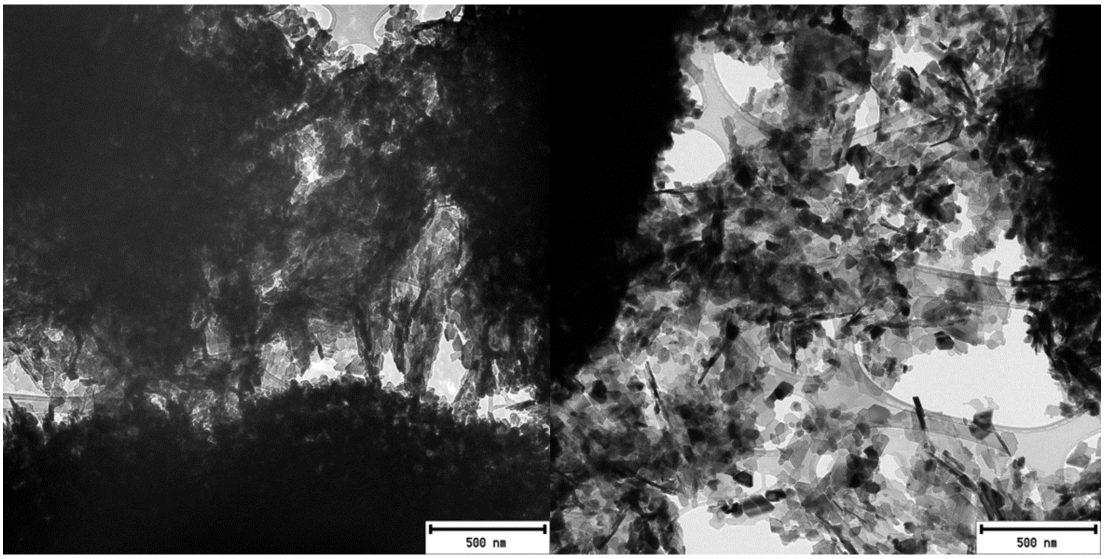

Supplementary Figure 5. *TEM images of agglomerated NTO nanosheets.*

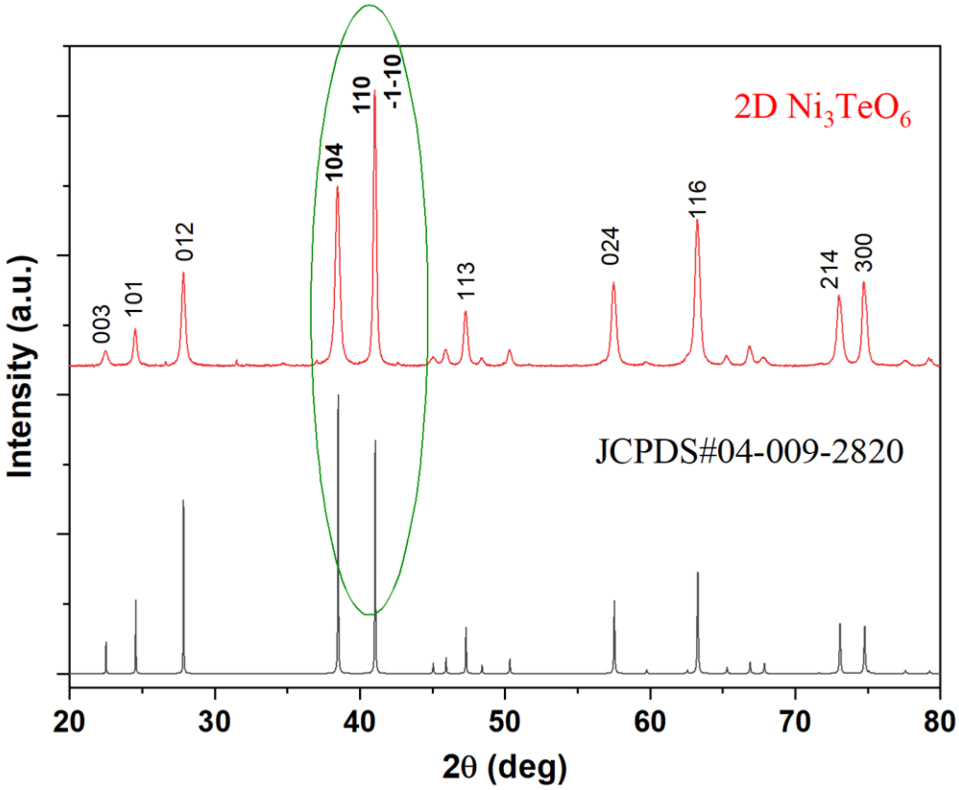

Supplementary Figure 6. *Comparative XRD plot for bulk vs 2D NTO.*

Comparative XRD plot for bulk vs 2D NTO showing preferred orientation in 2D NTO as (110) (-1-10) as they both planes share same 2 theta (41.04 ^{o}) position.

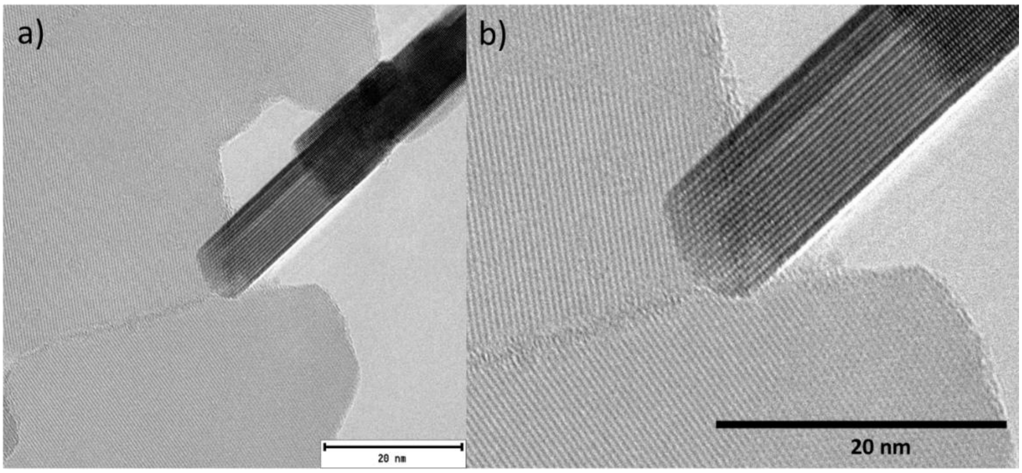

Supplementary Figure 7. *Morphology of NTO nanosheet.*

a) HR-TEM of 2D NTO and b) enlarged image of HR-TEM of 2D NTO

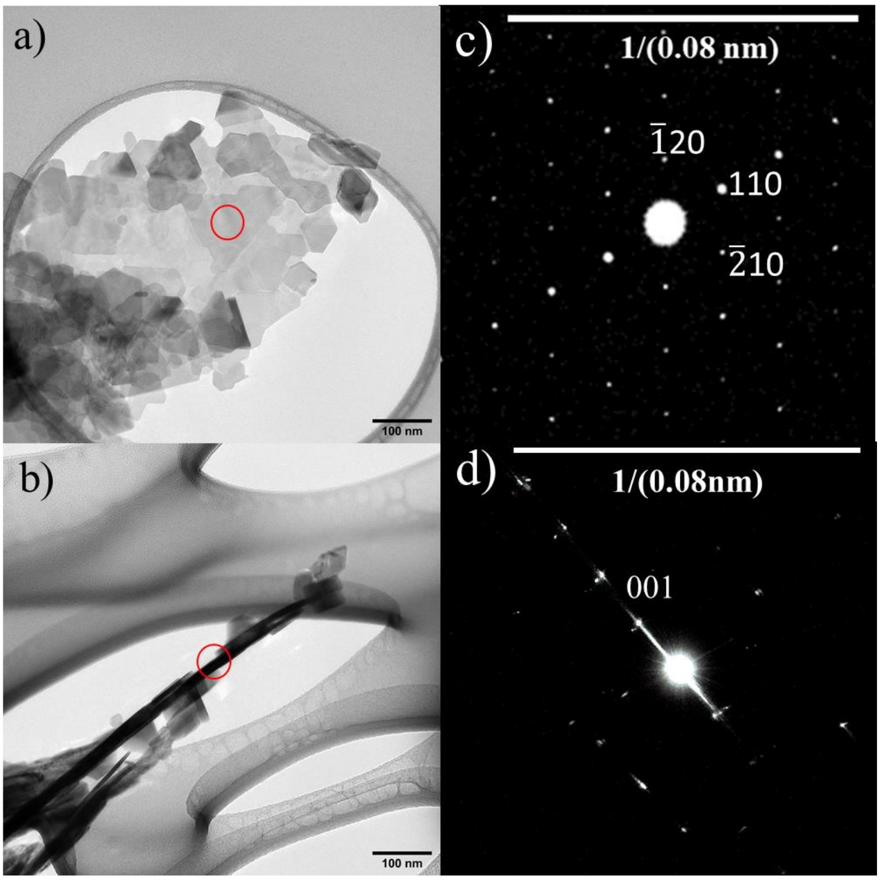

Supplementary Figure 8. *Morphology and SAED patter analysis of NTO nanosheets.*

(a-b) TEM and images of the 2D Ni_3TeO_6: (a) horizontal NTO, and (b) 90 degrees rotated NTO. (c-d) corresponding SAED patter of TEM images in (c), horizontal position and (d) vertically position, 90 degrees rotated.

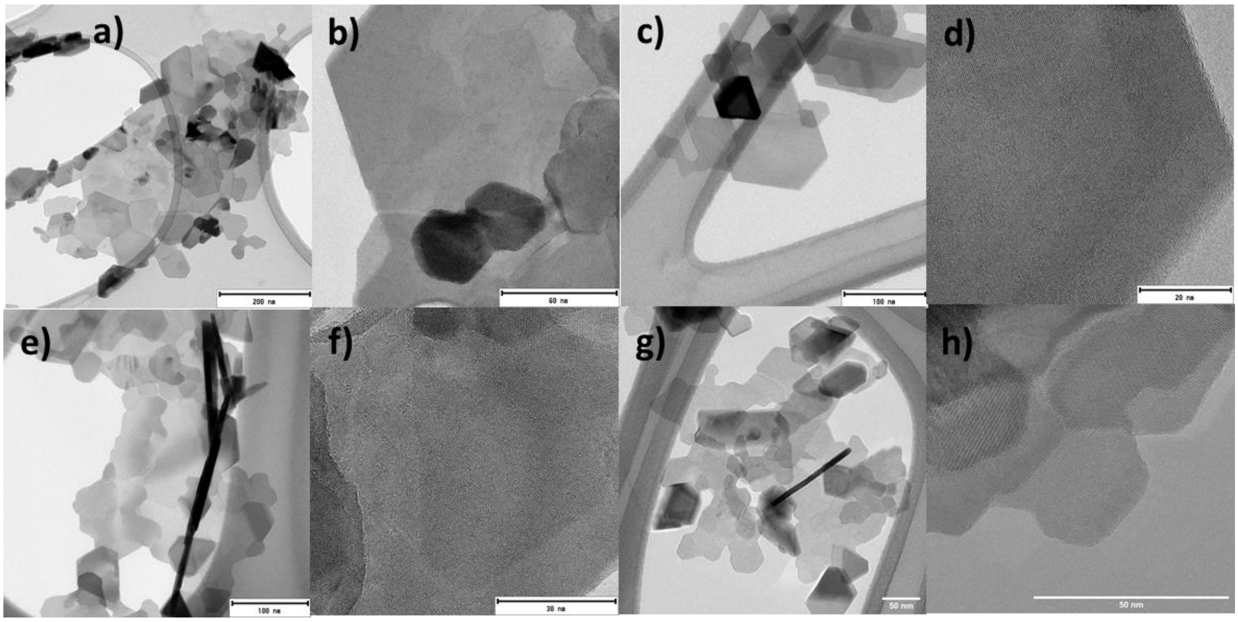

Supplementary Figure 9. *Stability analysis and reproduced 2D-NTO sample.*

(a) TEM image of the same 2D NTO sample analyzed for first time on 01.11.2021, (Fig 3), (b) HR-TEM images of the same 2D NTO powder analyzed for first time on 01.11.2021, (c) TEM image of the 2D NTO sample analyzed for second time on 02.02.2022, (d) H-RTEM image of the 2D NTO sample analyzed for second time on 02.02.2022, (e) TEM image of the 2D NTO sample analyzed for third time on 01.12.2022, (f) H-RTEM image of the 2D NTO sample analyzed for third time on 01.12.2022. (g) TEM image of the 2D NTO reproduced sample under the same preparation condition for repeatability confirmation, (h) HR-TEM image of the 2D NTO reproduced sample under the same preparation condition for repeatability confirmation.

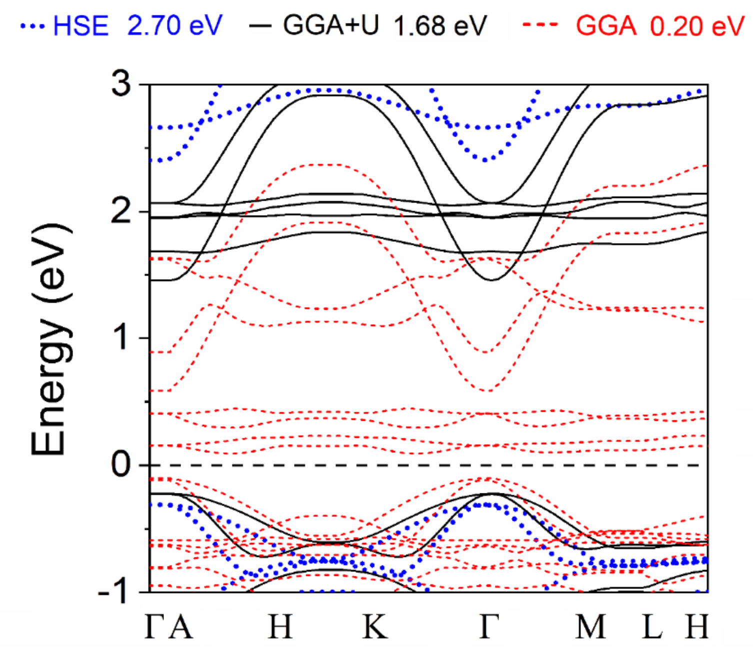

Supplementary Figure 10. *Theoretical band structure of 2D Ni_3TeO_6*

DFT predicted band structure of 2D Ni_3TeO_6 calculated using GGA, GGA+U, and HSE functionals. To achieve high precision in the calculation of the band structure of 2D NTO different functionals has been used. Despite the band structure of 2D NTO predicted by using GGA, GGA+U, and HSE functionals has similar shape, the GGA functional significantly underestimates the bandgap value which is 0.2 eV in that case. The best accuracy gives the HSE approach that gives the bandgap value of 2.70 eV for 2D NTO, which is large than that of obtained via GGA+U approach (1.68 eV). The value of U-J=2.5 eV for Ni was used in our calculations[1].

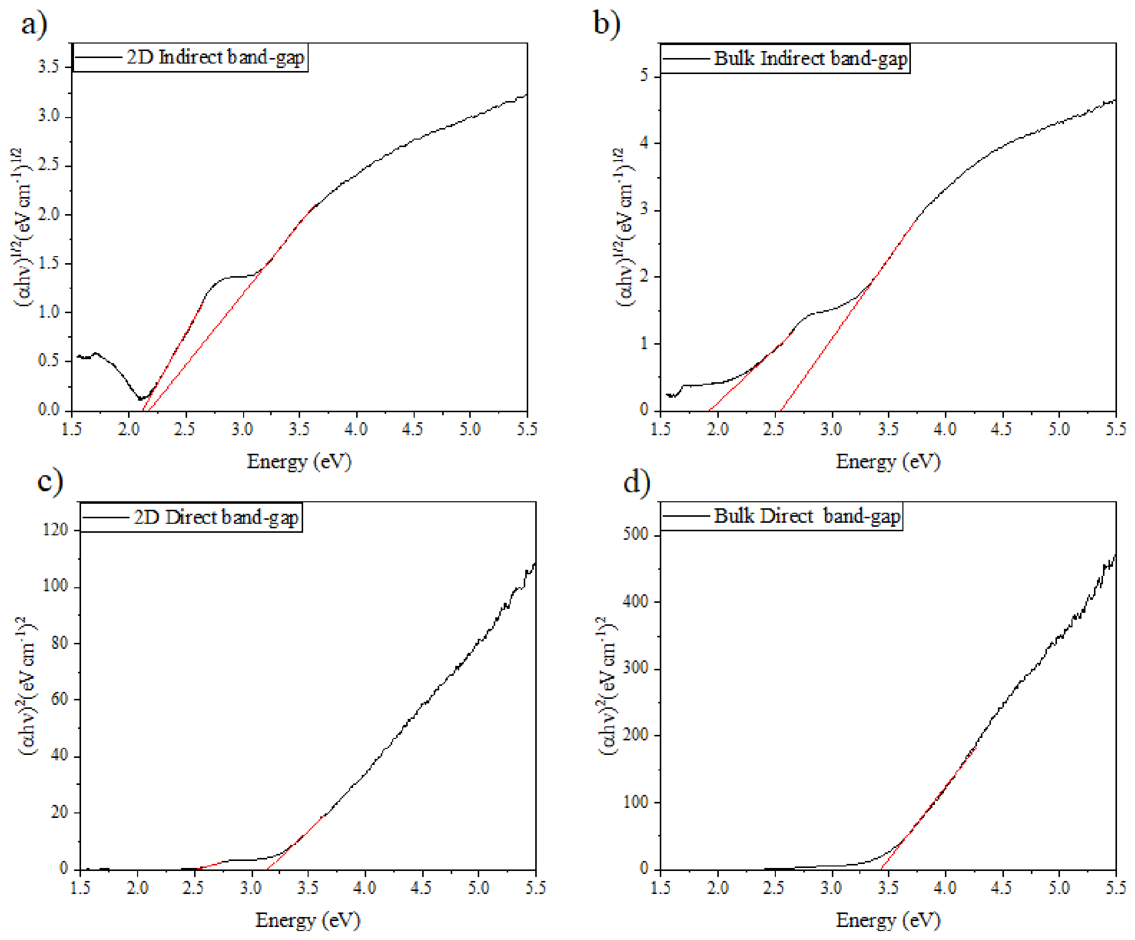

Supplementary Figure 11. *Experimental bandgap energy of Bulk and 2D NTO using UV-Vis spectroscopy.*

Bandgap energy (Eg) calculation from the Tauc plot (a) 2D NTO bandgap (b) Bulk NTO bandgap (c) 2D NTO bandgap, and (d) Bulk NTO bandgap.

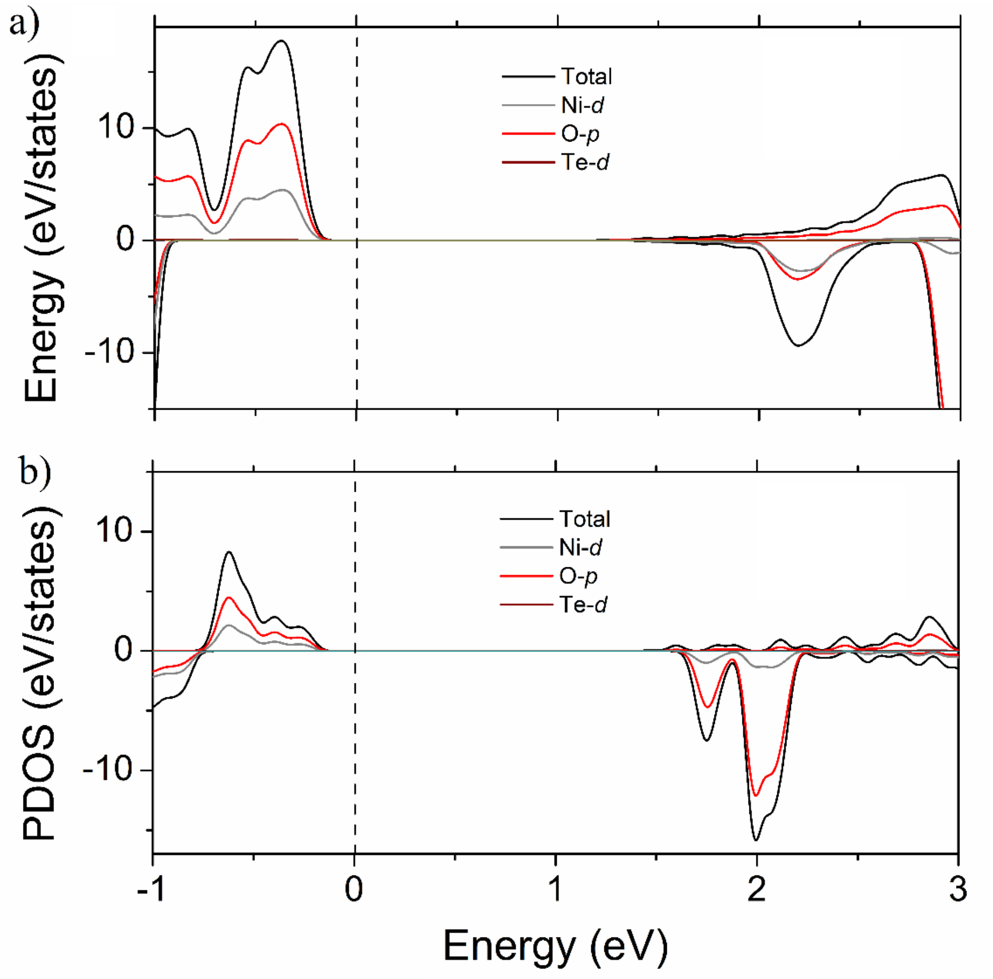

Supplementary Figure 12. *Partial density of states of 2D-NTO.*

PDOS of (a) 2D and (b) bulk NTO.

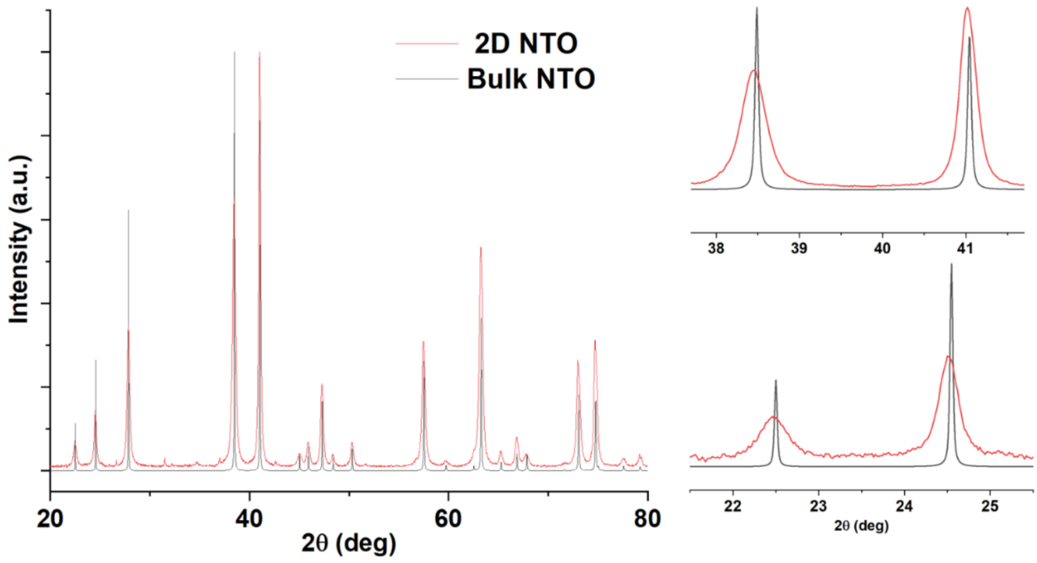

Supplementary Figure 13. *XRD result comparing 2D-NTO and Bulk materials***.**

XRD showing shift in peaks towards lower 2 theta side.

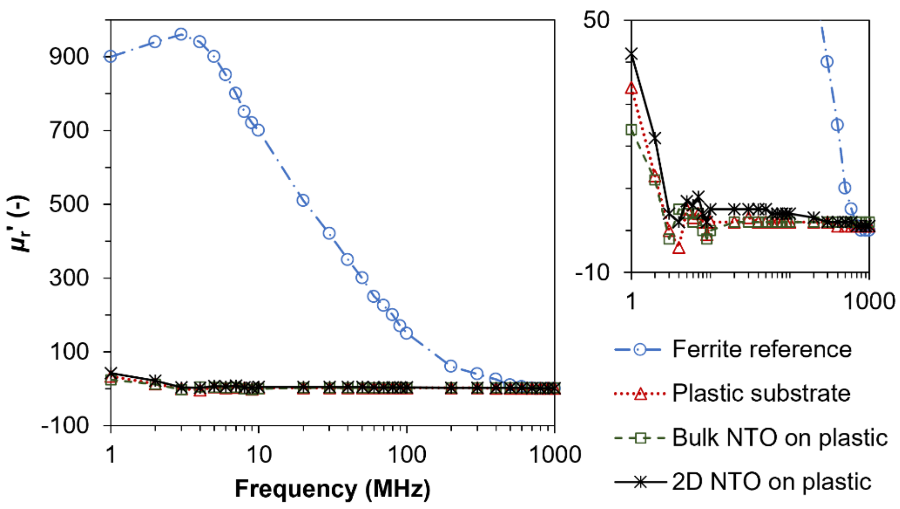

Supplementary Figure 14. *Magnetic properties of 2D-NTO material.*

The real part of the relative permeability (μ_r') measured at room temperature and in the range of 1 MHz – 1 GHz for the bulk and 2D NTO powders deposited on a plastic substrate as well as for a ferrite reference sample and the clean plastic substrate.

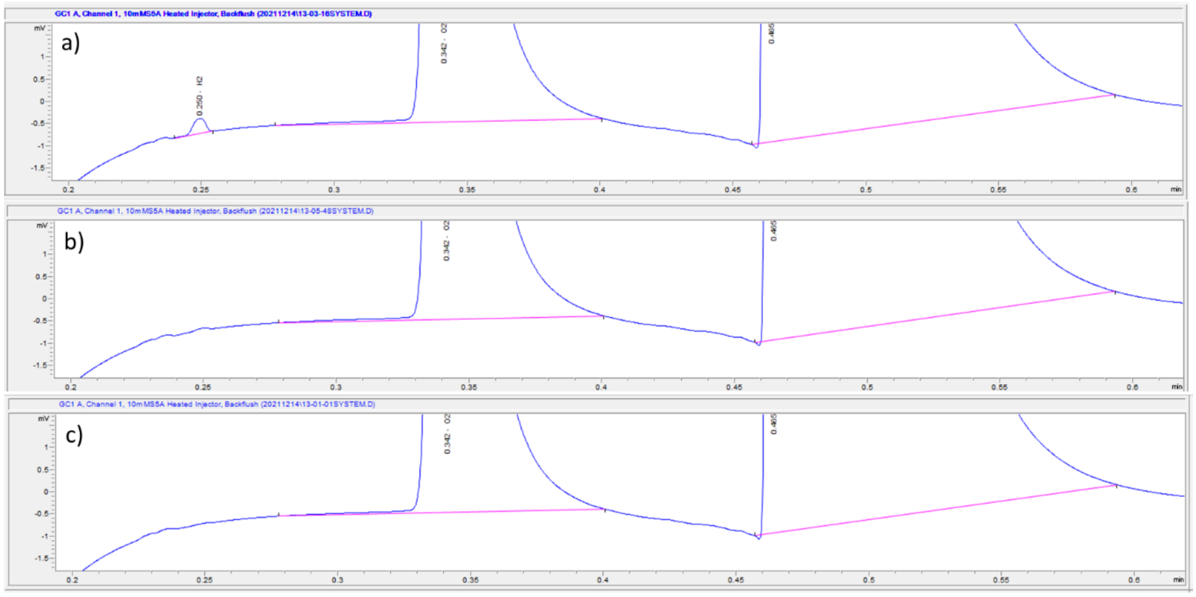

Supplementary Figure 15. *GC chromatograph of gases detected in H_2 evolution on Agilent Micro 490 GC (H_2 : 0.25 min, O_2: 0.342 min, and N_2: 0.465 min).*

a) 2D-NTO after 4 h of reaction, b) bulk-NTO after 4h of reaction, and c) Blank after 4h of reaction.

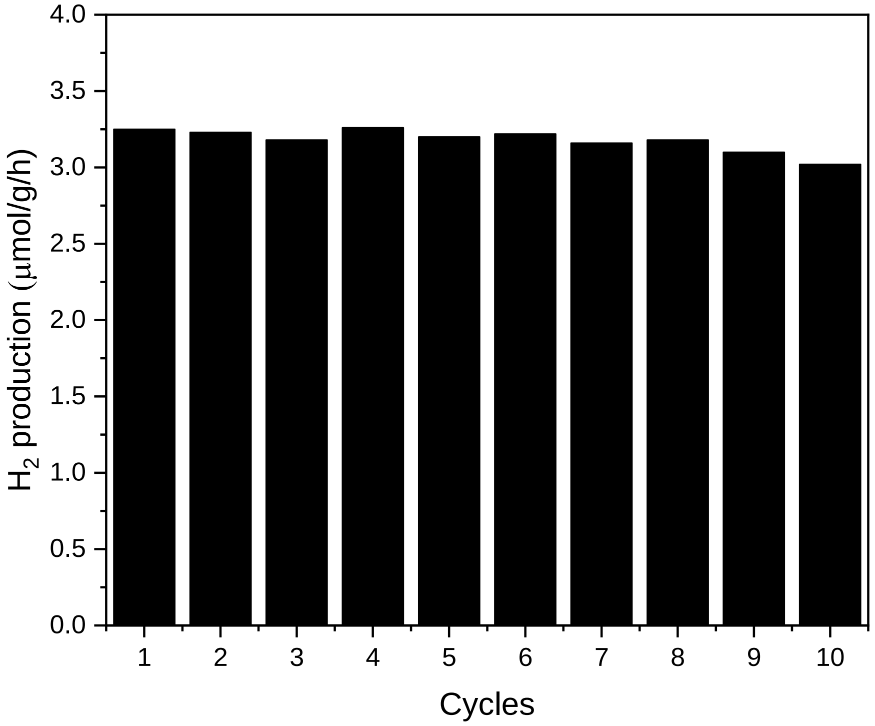

Supplementary Figure 16. *Re-cyclability of 2D NTO material for H_2 evolution*

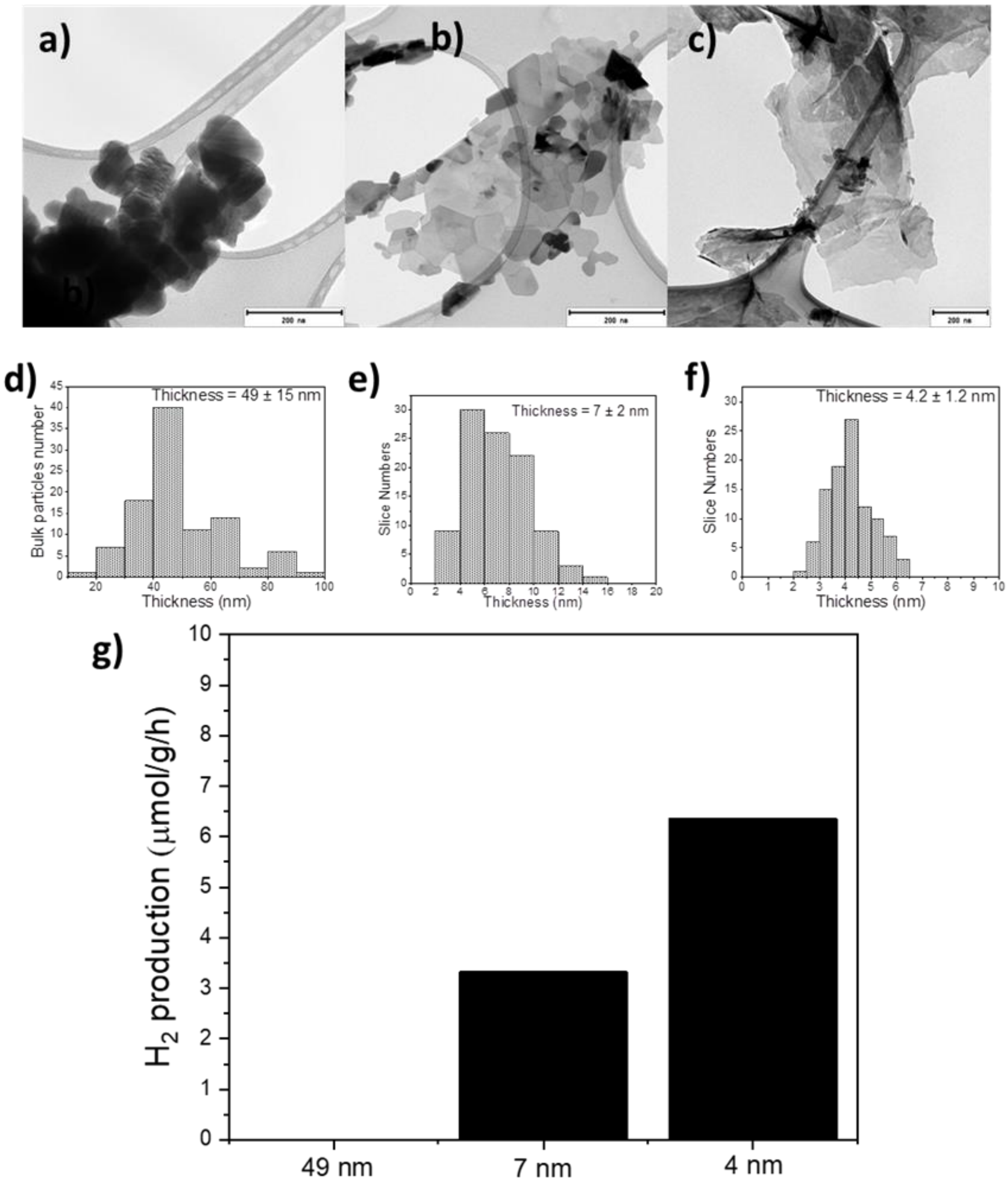

Supplementary Figure 17. *TEM images and photocatalytic activities of 2D NTO synthesised at different calcination conditions.*

(a) TEM image of the 2D NTO calcined at 5 ºC/min, (b) TEM image of the 2D NTO calcined at 8 ºC/min, (c) TEM image of the 2D NTO calcined at 10 ºC /min (d) average thickness of the nanosheets as statistically obtained for 2D NTO calcined at 5 ºC/min, (e) average thickness of the nanosheets as statistically obtained for 2D NTO calcined at 8 ºC/min. (f) average thickness of the nanosheets as statistically obtained for 2D NTO calcined at 10 ºC/min, (g) comparisons among photocatalytic activities of sample with different thickness.

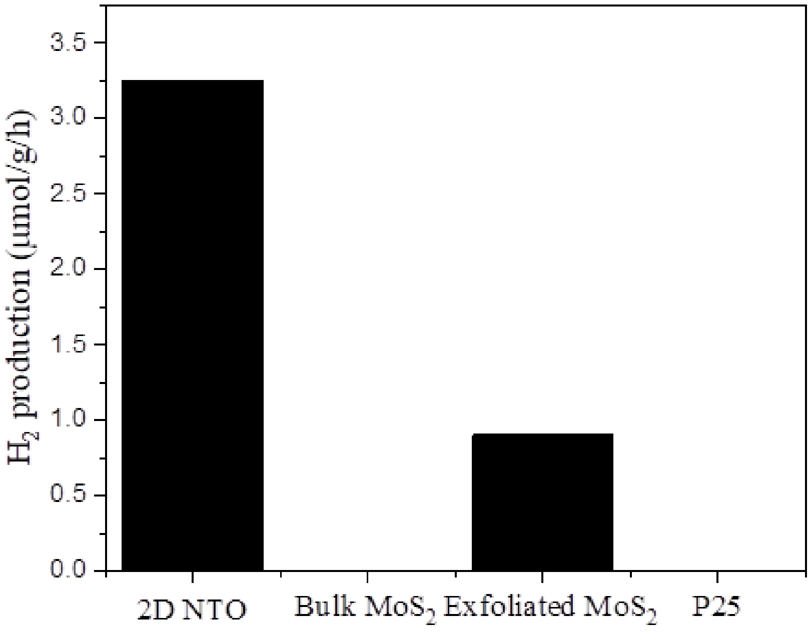

Supplementary Figure 18. *Catalytic activity in H_2 production of several pristine semiconductors.*

Comparisons among photocatalytic activities of 2D NTO, bulk MoS_2, exfoliated MoS_2 and P25 performed at the same conditions of reaction.

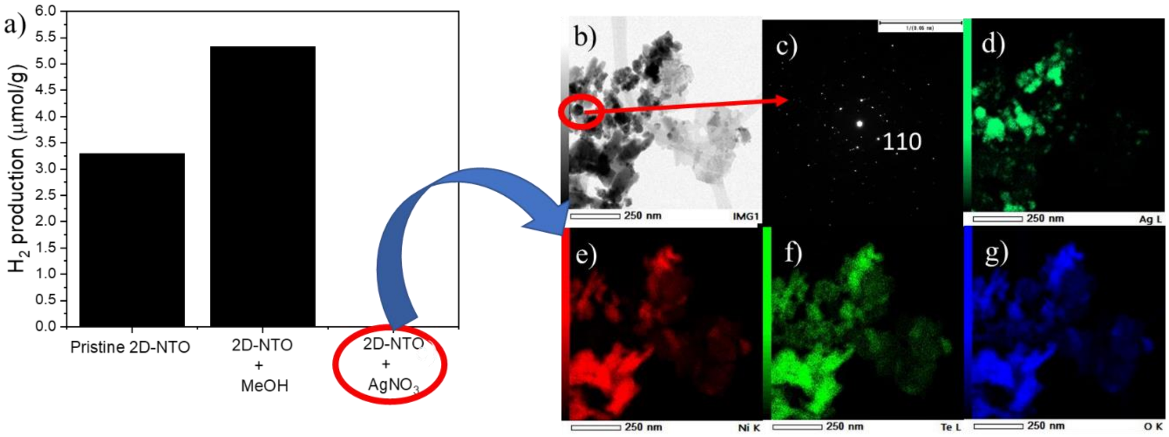

Supplementary Figure 19. *Scavenger effect in catalytic activity of 2D-NTO material.*

(a) Effect of electron and hole scavengers over 2D NTO. (b) STEM image of 2D NTO after use $AgNO_3$ as electron scavenger in HER. (c) Corresponding SAED patter of one silver metallic particle (110) of 2D-NTO +$AgNO_3$ sample. (d-g) corresponding EDS mapping of STEM image (b), elemental images of d) Ag, e) Ni, f) Te, g) O.

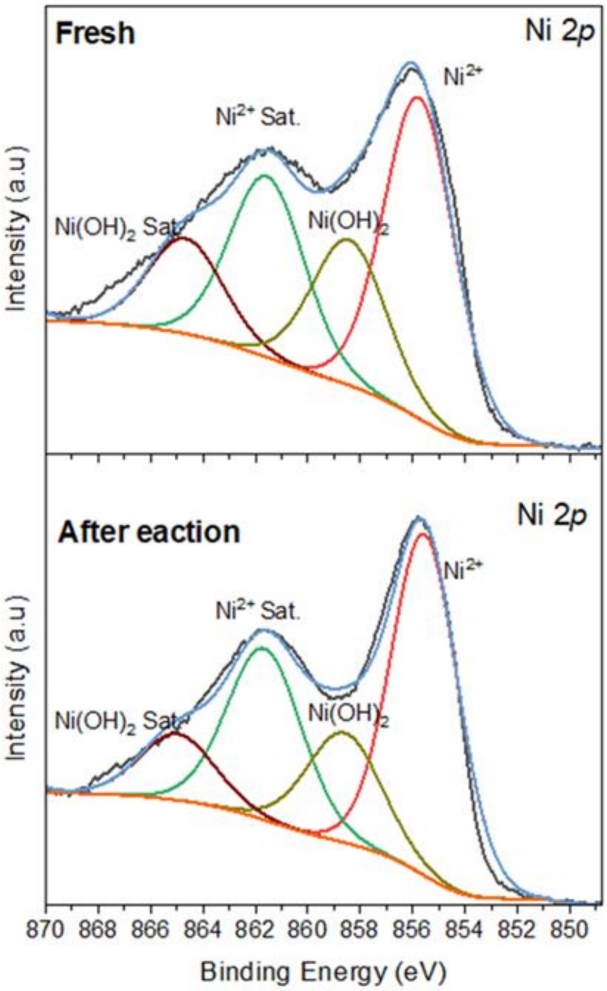

Supplementary Figure 20. *Chemical composition of 2D-NTO before and after reaction. XPS spectra of Ni 2P from fresh 2D sample and after reaction 2D sample.*

Supplementary Table

Supplementary Table 1. Comparison of H_2 production activity over 2D pristine photocatalysts without sacrificial agent and in visible light.

**Samples**	**Synthesis methodology**	**H_2 Production Activity ($\mu mol\ g^{-1}\ h^{-1}$)**	**Reference**
2D NTO	Hydrothermal	3.2	**This work**
MoS_2	Exfoliation	0.9	Our group
Pure g-CN	Thermal heating	<0.1	2
$BiVO_4$ nanosheets	Hydrothermal + sonication	<0.2	3
TiO_2 nanosheets	Hydrothermal	0	4
CdS	Hydrothermal	1.5	5
MoS_2	Hydrothermal	0	6
ZnCdS	Hydrothermal	0	7
Co@CoO	Hydrothermal	0	7

Supplementary References.

1. Long, O. Y. et al. Evaluating optimal U for 3d transition-metal oxides within the SCAN+U framework. *Phys. Rev. Mater.* **4**, 045401 (2020).

2. Zhang, G. et al. Iodine Modified Carbon Nitride Semiconductors as Visible Light Photocatalysts for Hydrogen Evolution. *Adv. Mater.* **26**, 805-809 (2014).

3. Li, Y. et al. 2D/2D heterostructure of ultrathin $BiVO_4/Ti_3C_2$ nanosheets for photocatalytic overall Water splitting. *Appl. Catal. B.* **285**, 119855 (2021).

4. Liu, X. et al. Engineering TiO_2 nanosheets with exposed (001) facets via the incorporation of Au clusters for boosted photocatalytic hydrogen production. *Mater. Adv.* **1**, 1608-1612 (2020).

5. Ai, Z. et al. Construction of CdS@Ti_3C_2@CoO hierarchical tandem p-n heterojunction for boosting photocatalytic hydrogen production in pure water. *Chem. Eng. J.* **383**, 123130 (2020).

6. Zhang, Z. -W. et al. One-pot hydrothermal synthesis of willow branch-shaped MoS_2/CdS heterojunctions for photocatalytic H_2 production under visible light irradiation. *Chinese J. Catal.* **40**, 371–379 (2019).

7. Zou, Y. et al. Photocatalytic performance and mechanism of hydrogen evolution from water over ZnCdS/Co@CoO in sacrificial agent-free system. *Int. J. Hydrog. Energy*, **47**, 25289-25299 (2022).
